# High NESTIN Expression Marks the Endosteal Capillary Network in Human Bone Marrow

**DOI:** 10.3389/fcell.2020.596452

**Published:** 2020-12-08

**Authors:** Francesca M. Panvini, Simone Pacini, Marina Montali, Serena Barachini, Stefano Mazzoni, Riccardo Morganti, Eugenio M. Ciancia, Vittoria Carnicelli, Mario Petrini

**Affiliations:** ^1^Institute of Life Sciences, Sant’Anna School of Advanced Studies, Pisa, Italy; ^2^Department of Clinical and Experimental Medicine, University of Pisa, Pisa, Italy; ^3^Department of Translational Research and New Technology in Medicine, University of Pisa, Pisa, Italy; ^4^Statistical Support to Clinical Trials Department, Azienda Ospedaliero Universitaria Pisana, Pisa, Italy; ^5^Department of Pathology, Azienda Ospedsaliero Universitaria Pisana, Pisa, Italy; ^6^Department of Surgical, Medical and Molecular Pathology and Critical Care Medicine, University of Pisa, Pisa, Italy

**Keywords:** bone marrow, micro-vessels, niche, NESTIN, hematopoietic stem cells, endosteum, capillaries, CD146

## Abstract

Hematopoiesis is hosted, supported and regulated by a special bone marrow (BM) microenvironment known as “niche.” BM niches have been classified based on micro-anatomic distance from the bone surface into “endosteal” and “central” niches. Whilst different blood vessels have been found in both BM niches in mice, our knowledge of the human BM architecture is much more limited. Here, we have used a combination of markers including NESTIN, CD146, and αSMA labeling different blood vessels in benign human BM. Applying immunohistochemical/immunofluorescence techniques on BM trephines and performing image analysis on almost 300 microphotographs, we detected high NESTIN expression in BM endothelial cells (BMECs) of small arteries (A) and endosteal arterioles (EA), and also in very small vessels we named NESTIN^+^ capillary-like tubes (NCLTs), not surrounded by sub-endothelial perivascular cells that occasionally reported low levels of NESTIN expression. Statistically, NCLTs were detected within 40 μm from bone trabecula, frequently found in direct contact to the bone line and spatially correlated with hematopoietic stem/progenitor cells. Our results support the expression of NESTIN in human BMECs of EA and A in accordance with the updated classification of murine BM micro-vessels. NCLTs for their peculiar characteristics and micro-anatomical localization have been here proposed as transitional vessels possibly involved in regulating human hematopoiesis.

## Introduction

Hematopoietic stem/progenitor cells (HSPCs) are a highly dynamic multicellular system, which gives rise to all types of fully functional mature blood cells. During adulthood, HSPCs mainly reside within a specific bone marrow (BM) microenvironment usually referred to as “niche” (Schofield, 1978), which influences the HSPC fate by intrinsic molecular programs and extrinsic signals (Yu and Scadden, 2016). Several authors described two main niche components distinctly located in the endosteal and perivascular regions (Doan and Chute, 2012; Al-Drees et al., 2015). However, such a distinction has been questioned (Lo Celso et al., 2009; Beerman et al., 2017) due to the complex endosteal microvascular network sustained by arteries and arterioles. Recent findings in mice models showed differences between the arterial/arteriolar network and the sinusoidal plexus in perivascular niches (Spencer et al., 2014; Itkin et al., 2016). Morphology, presence of a sub-endothelial layer of perivascular cells, blood flow velocity, permeability, and differential expression of specific markers in bone marrow endothelial cells (BMECs), allowed to distinguish arterial/arteriolar micro-vessels from sinusoidal. Recently, among those differentially expressed markers, NESTIN attracted particular attention.

NESTIN was originally described as a 176 kDa class VI intermediate filament protein (Guérette et al., 2007) in neural stem cells of embryonic and adult brain (Lendahl et al., 1990; Mignone et al., 2004; Di et al., 2014). Later, NESTIN expression was evidenced in a many cell types, tissues, and organs, including endothelial cells (Suzuki et al., 2010), fibroblasts (Kishaba et al., 2010), heart (Liu et al., 2015), dental tissue, testes, hair follicles, skin, pancreas, and newly formed blood vessels (Zulewski et al., 2001; Wiese et al., 2004; Du et al., 2011; Abe et al., 2012), and its reported involvement in cell proliferation and division (Dupin and Etienne-Manneville, 2011) supports the hypothesis that NESTIN may be required for stem/progenitor cell self-renewal and differentiation in several tissues (Bernal and Arranz, 2018). NESTIN is also considered having a role during angiogenesis. In rat fetuses at the early stage of development (E14-15), NESTIN has been detected in endothelial cells in all blood vessels, while such expression was not reported in adult ECs (Matsuda et al., 2013). NESTIN may also play an important role in angiogenesis of wound healing in different tissues and organs, such as pancreas (Ishiwata et al., 2006), skin (Aki et al., 2010) and heart (Calderone, 2012).

In *Nestin-gfp* transgenic mice, Méndez-Ferrer et al. (2010) showed that NESTIN expression can be used to identify HSPC-niche forming mesenchymal stromal cells (MSCs). The NESTIN^+^ cell fraction included fibroblastic colony-forming units (CFU-Fs), which retained tri-lineage differentiation, robust self-renewal, and directly influenced HSPC maintenance and BM homing. Subsequently, Kunisaki et al. (2013) described two distinct subsets of NESTIN^+^ perivascular cells: rare *Nestin-gfp*^*bright*^ cells surrounding arterioles and reticular *Nestin-gfp*^*dim*^ cells in close contact with sinusoids. Pinho et al. (2013) showed that the expression of Platelet-Derived Growth Factor-α (PDGFRα) and CD51 (Integrin α-V) surface markers characterizes a large fraction of NESTIN^+^ cells in *Nestin-gfp*^+^ mice. Interestingly, the two novel markers were able to identify NESTIN^+^ stromal cells also in human fetal BM (Pinho et al., 2013). PDGFRα^+^/CD51^+^ cells represent a small subset of the population positive for CD146, previously identified as a human pericyte marker (Crisan et al., 2008). Nonetheless, Isern et al. (2014) and Itkin et al. (2016) described NESTIN expression also in Sca-1^+^ VE-cadherin^+^ CD31^*high*^ arterial BMECs (aBMECs) but not in sinusoidal (sBMECs). Associated with calcified bone, these NESTIN-positive, di-acetyl low-density lipoprotein (LDL)-negative aBMECs resulted in more actively developing ECs.

Despite a consistent number of observations in animal models, NESTIN expression is still poorly investigated in humans. Dusart et al. (2018) recently reported data from transcriptome and antibody-based analysis of tissue microarrays (TMA) form adult human organs. This study revealed body-wide NESTIN expression in the ECs, both in normal and tumor tissues, independently from their proliferative status and debating the idea of NESTIN as neo-vascularization associated marker (Suzuki et al., 2010; Matsuda et al., 2013). Moreover, the gene ontology (GO) analysis of 150 transcripts showed the relationship between *NES* and the EC-enriched transcripts such as *TIE1*, *TEK*, and *CD34*. However, results about NESTIN expression in BMECs are not clearly reported and convincing, compared to other tissues. In fact, the representativeness of BM TMA could strongly be affected by its tissue complexity and heterogeneity, even having the whole organ available. Particularly in humans, the selection of the core from BM trephines based on cellularity could exclude important regions i.e., those rich in adipocytes and/or bone trabecula (Tjin et al., 2019).

Here we have characterized different human BM vessels using different markers including NESTIN. We have used an approach different from TMA, whereby NESTIN has been detected to localize and characterize aBMECs in the whole area of sections from a consistent number of different BM benign biopsies. The anatomical and functional distinction of BM blood vessels, deeply investigated in last years in murine models, paves the way for a more precise definition of the mechanisms that regulate the interaction of HSPCs and their niches in humans. Gaining knowledge of the reciprocal regulation of the hematopoietic and non-hematopoietic compartments will thus have important implications in understanding stem cell fate in health and disease, and could be relevant for stem cell mobilization and transplantation protocols in clinics.

## Materials and Methods

Slides were obtained from de-identified archival paraffin-embedded BM biopsies showing normal histology, hereinafter referred as “benign biopsies.” BM trephines were obtained from the iliac crest of 17 patients (11 male and 6 female, median age: 57), 13 staged for lymphoma (2 Hodgkin’s and 11 non-Hodgkin’s) and four evaluated for cytopenia. Sections of 3 μm were processed for immunohistochemistry to detect NESTIN. In parallel, Immunofluorescence was carried out applying anti-human NESTIN, anti-human CD146, anti-human CD34, anti-human alpha Smooth Muscle Actin (αSMA), anti-human ENDOMUCIN (EMCN), anti-human CD31 and anti-human von Willebrand factor (vWF) primary antibodies (Abcam, Cambridge, United Kingdom). Slides were mounted in anti-fade reagent with 4′,6-diamidino-2-phenylindole (DAPI) (Thermo Scientific) for nuclei detection.

Pictures showing NESTIN^+^ structures were included in the study and processed to measure vessel inner caliber (IC), number of CD34^+^ cells and their distance (Supplementary Figure 1). The presence of CD146^+^ or αSMA^+^ perivascular cells was also recorded. Distances were normalized by subtracting half of the IC. When applicable, vessel distance from trabecula was also recorded (Supplementary Figure 1). Due to their negative stain to NESTIN and CD146, sinusoids were excluded by the immunohistological classification of BM micro-vessel, here reported, even if important regulators of HSPCs and hematopoiesis.

NESTIN, CD34, and CD31 Co-localization analysis was performed applying Costes’ auto-threshold (Costes et al., 2004), Pearson’s and Mander’s coefficient values were recorded for pixels above the threshold. Co-localization negative control images were obtained translating the NESTIN channel with 100 pixels offsets in both on *X*- and *Y*-axis.

“Forced co-labeling” was also performed obtaining four-color images for simultaneous non-quantitative detection of NESTIN, CD34, and CD146 (Supplementary Figure 2). More detailed methods in Supplementary Material 1.

Histomorphometric data regarding inner caliber, presence of perivascular cells and tubular shape were analyzed in a 5 × 3 contingency table. The dichotomous values “presence/absence of perivascular cells” and “tubular/no tubular shape” were combined with the caliber measures divided into quartiles, the *chi-square* test was applied. The distribution of data regarding distance from the bone line and HSPCs were evaluated by graphical method (Q-Q plot chart) and normality was analyzed by the Shapiro–Wilk test. Comparisons between of co-localization mean coefficients were performed applying Kruskal–Wallis ANOVA test and Dunns’ post-test. All analyses, descriptive and inferential, were carried out by SPSS v.25 technology.

## Results

### Immunohistochemistry Revealed Distinct NESTIN-Positive Micro-Vessels and Sporadic Cells

**FIGURE 1:**
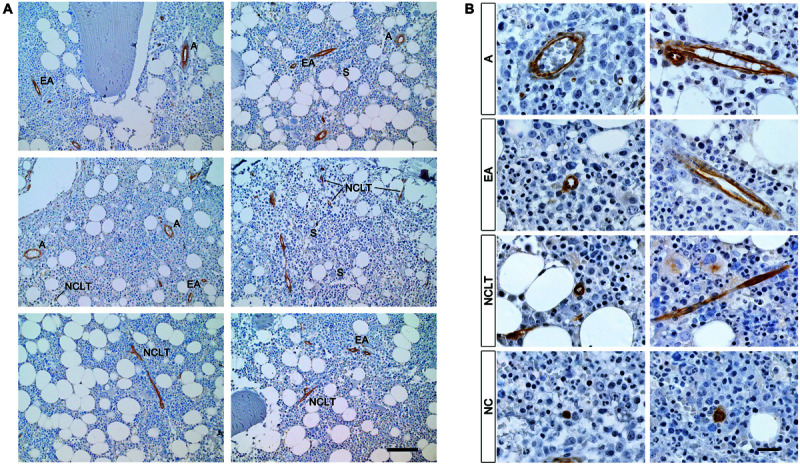
NESTIN^+^ vascular structures in human benign bone marrow. **(A)** Immunoperoxidase reaction revealed NESTIN expression in BMECs of arteries (*A*) and endosteal arterioles (*EA*) while sinusoids (*S*) showed negative. Positive NESTIN stain also revealed tubular vessels characterized by very small inner caliber and absence of NESTIN-positive perivascular cells we referred to as NESTIN^+^ capillary-like tubes (NCLT). Scale bar = 200 μm. **(B)** Under high power magnification, the intense NESTIN stain was confirmed in BMECs of arteries (*A*) and endosteal arterioles (*EA*). Some perivascular cells, showing elongated nuclei, in the perivascular region of *A* and *EA* showed weak NESTIN expression. Intense positivity was also detected in endothelial cells of capillary-like tubes (*NCLT*). NCLT capillary-like nature was confirmed by very high magnification power (1,000×) that showed lack of perivascular NESTIN-positive cells and presence of many erythrocytes compressed within the vessel walls. Isolated NESTIN^+^ cells or small clusters (2–3 cells) (*NC*) were also detected at high frequency. Scale bar = 20 μm.

Under low power magnification (200×), resulting in a field of view (FOV) of 1.25 mm in diameter (Φ), vascular and microvascular structures including arteries, arteriolar vessels and capillary-like tubes were NESTIN^+^. In contrast, sinusoids (S, larger vessels with thin endothelial layer, convolute structure, and extended lumen area) were NESTIN^–^ (Figure 1A and Supplementary Figure 3A). Following the recent description of murine BM vessels by Itkin et al. (2016), we classified NESTIN^+^ structures in arteries (A), characterized by lumen diameter over 2–3 cell size, elongated morphology of the endothelial cell nuclei and a complex second layer of perivascular cells; or smaller endosteal arterioles (EA) with a diameter in the order of 1 cell size. Besides, very small capillary-like tubular structures, not ascribable to A or EA due to their small caliber and apparent lack of a clear subendothelial layer of perivascular cells, were frequently detected for their positive stain for NESTIN. These structures showed imperceptible lumens and were described as NESTIN-positive capillary-like tubes (NCLT).

Under high power magnification (1,000×, FOV Φ = 0.25 mm) both As and EAs confirmed NESTIN expression albeit with different levels. Whilst high NESTIN expression was found in BMECs, a comparatively lower NESTIN expression was detected in perivascular cells, which showed elongated nuclei (Figure 1B). The latter has been confirmed by a positive correlation of mRNA expression for *NES* and endothelial-associated genes, such as *PECAM1* (CD31, *R* = 0.907, *p* < 0.05) and *CD34* (*R* = 0.905, *p* < 0.05, Supplementary Figure 8A and Supplementary Data 2), as well as the MSC-associated markers *ALCAM* (CD166, *R* = 0.925, *p* < 0.05) and *NT5E* (CD73, *R* = 0.905, *p* < 0.05, Supplementary Figure 8B). Nonetheless, the expression of *NES* in purified BMECs resulted in 3-log higher compared with sorted MSCs (*p* < 0.01, Supplementary Figure 8C).

NCLT capillary-like nature was often suggested by the presence of compressed erythrocytes in lumens, which could be appreciated both in transverse and longitudinal sections (Supplementary Figure 4). Isolated NESTIN^+^ cells or small clusters (2–3 cells) were also observed (NCs, Figure 1B and Supplementary Figure 4). Interestingly, both NCLTs and NCs were frequently identified in close proximity to bone trabecula (Supplementary Figure 3B).

### Spatial Co-occurrence of NESTIN and Endothelial Markers

**FIGURE 2:**
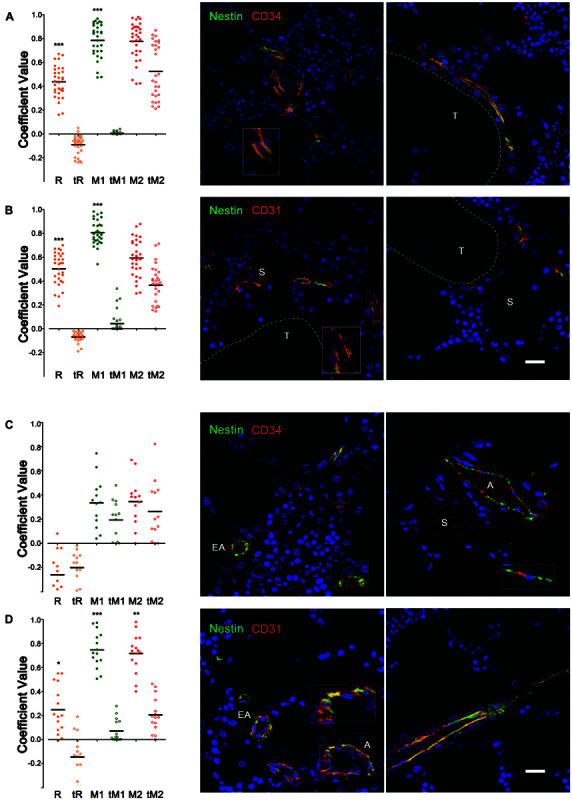
Co-occurrence of NESTIN and endothelial markers in bone marrow vessels. Co-localization analysis of **(A)** NESTIN and CD34 expression in NCLTs revealed a positive correlation with significant higher Pearson’s coefficient (R) respect to the translated negative control (*t*R). Intensity independent Mander’s coefficients showed the almost perfect co-localization of CD34 in the NESTIN channel (M1 respect to *t*M1), together with no significant co-localization of NESTIN in the CD34 channel (M2 respect to *t*M2), supporting the “*co-occurrence*” of these two markers that occupy different cell compartment, where NESTIN (green) is cytoskeleton associated protein while CD34 (red) is a cell surface marker. **(B)** Similar results were obtained analyzing expression of NESTIN and CD31. **(C)** Co-localization analysis of NESTIN and CD34 expression in *A*s and *EA*s did not reveal any correlation with Pearson’s (R) and Mander’s coefficients (M1, M2) similar to the translated negative control (*t*R, *t*M1, and *t*M2). Indeed, red and green signals resulted spatially segregated in the endothelial vessel walls. Conversely, **(D)** significant co-occurrence of NESTIN and CD31 were reported, where the NESTIN (green) and CD31 (red) signals overlapped (yellow) in a good portion of the endothelial vessel’s wall. DAPI stain in blue (S = sinusoid, T = bone trabecula, scale bar = 20 μm, *p < 0.05, **p < 0.01, ***p < 0.001).

To support the hypothesis of the endothelial expression of NESTIN in human BM vessels, the co-localization analysis was performed on confocal microphotographs where channel 1 (Ch1) reported expression of NESTIN (green pseudocolor) and channel 2 (Ch2) the expression of CD34 or CD31 (red pseudocolor). Analyzing NCLTs, a significant positive correlation was detected between NESTIN and CD34 expression with a mean Pearson’s coefficient (*R*) = 0.437 ± 0.024 compared with the negative control (*tR* = −0.091 ± 0.014, *p* < 0.001 *n* = 31, Figure 2A). Because NESTIN is a cytoskeletal protein, whilst CD34 is a transmembrane protein, a perfect co-localization (*R* ≈ 1) was not expected for these two molecules with different subcellular distribution (Figure 2A, magnified view in the box). Indeed, Mander’s analysis (Manders et al., 1993) demonstrated the co-localization of CD34 and NESTIN with the mean coefficient *M1* = 0.785 ± 0.026 significantly higher than control (*tM1* = 0.006 ± 0.002, *p* < 0.001, *n* = 31) and close to 1, while the *M2* and *tM2* were not significantly different, demonstrating a co-occurrence of NESTIN with the CD34 endothelial marker in NCLTs. Similar results were also obtained after analyzing the co-occurrence of NESTIN and a different endothelial surface marker, such as Platelet-endothelial Cell Adhesion Molecule 1 (CD31), with even higher mean correlation coefficients: *R* = 0.503 ± 0.025 vs. *tR* = −0.069 ± 0.007 and *M1* = 0.804 ± 0.018 vs. *tM1* = 0.043 ± 0.016 (Figure 2B). However, in As and EAs co-occurrence of NESTIN and CD34 was not detected (Figure 2C), most likely due to the possible presence of mesenchymal CD34^–^ NESTIN^+^ subendothelial cells in these vessels and probably influenced by a non-overlapping subcellular expression pattern of NESTIN and CD34 in endothelial cells (Figure 2C). Supporting the latter, NESTIN and CD31 significantly co-occurred in A/EAs (*R* = 0.249 ± 0.051 vs. *tR* = −0.144 ± 0.048, *p* < 0.050; *M1* = 0.746 ± 0.038 vs. *tM1* = 0.072 ± 0.024, *p* < 0.001 and *M2* = 0.717 ± 0.042 vs. *tM2* = 0.206 ± 0.038, *p* < 0.010, *n* = 15, Figure 2D). These results have been also supported by flow cytometry data demonstrating the existence of a small BM cell population (0.46 ± 0.12% of BM cells) co-expressing CD34, CD31 and NESTIN (Supplementary Data 2 and Supplementary Figure 8D). These data indicate that, resembling previous findings in mouse (Isern et al., 2014; Itkin et al., 2016), NESTIN marks endothelial and sub-endothelial cells in A and EAs. Additionally, the results reveal the presence of endosteal capillaries with high NESTIN expression (NCLTs).

### Extended Morphological Classification of NESTIN-Positive Micro-Vessels

**FIGURE 3:**
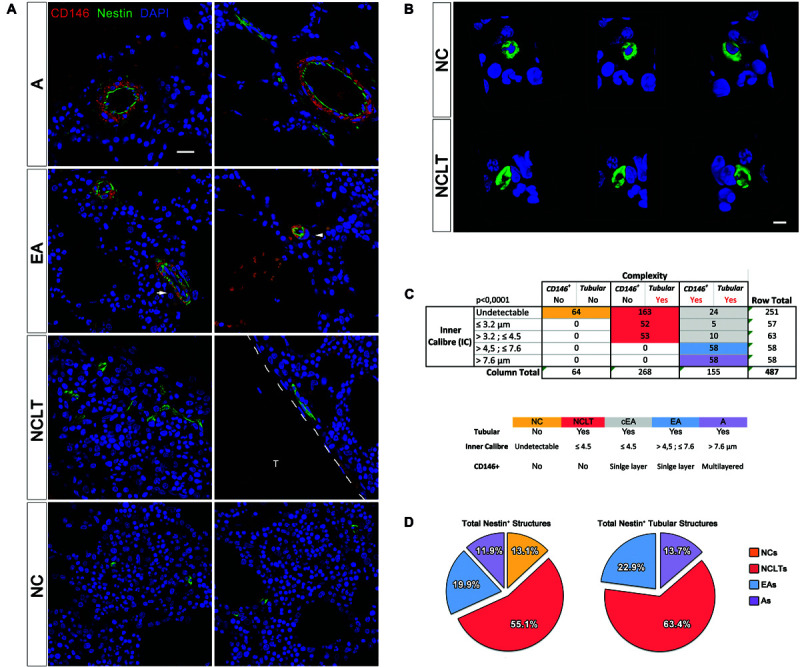
Classification and quantification of NESTIN^+^ vascular structures. **(A)** Single optical sections allowed accurate measurement of the inner caliber (IC) of NESTIN^+^ (green) structures and the correlation to the CD146 (red) perivascular cells (scale bar = 20 μm, T = trabecula). **(B)** Due to the tiny lumens, several transversal sections of capillaries could be confused with single NESTIN^+^ cells. In these cases, a 3D-reconstruction was applied to discriminate single cells (NC) showing spheroidal and “no-tubular” NESTIN^+^ (green) structure surrounding a DAPI^+^ (blue) nucleus. NESTIN^+^ capillary-like tubes (NCLT) revealed their “tubular” nature, showing the hollow fiber-like appearance (scale bar = 5 μm). **(C)** The contingence (*p* < 0.001) has been analyzed by a 5 × 3 table, including IC quartiles and complexity. We grouped NESTIN^+^ structures in arteries (IC >7.2 μm, complexity: Y/Y, purple box), arterioles (4.6–7.2 μm, complexity: Y/Y, pale blue box) and capillaries (IC <4.6 μm, complexity: N/Y). Sub-endothelial perivascular cells were also detected in about 30% of <4.6 μm micro-vessels, which we believe to be arterioles with apparent IC <4.6 μm due to compression or sectioning artifacts [white arrow in panel **(A)**, gray box in panel **(B)**]. Confocal images showing segregated expression of NESTIN (green) and CD146 (red) in arterial BMEC and sub-endothelial adventitia of bone marrow arteries, respectively. EA showed selective NESTIN/CD146 expression in more interdigitating cells or cells encircled by single pericytes (white arrowhead). **(D)** NCLTs resulted the most frequent NESTIN^+^ microvascular structures.

We further classified BM NESTIN^+^ cells using multicolor confocal images (*n* = 297; an average of 18 per sample) and the additional marker CD146, which marks endothelial and subendothelial MSCs in human BM (Sacchetti et al., 2007; Figure 3A). Taking into account their inner caliber (IC), NESTIN^+^ vessels (*n* = 487) were classified into five groups with cut-offs defined by 25^th^, 50^th^, and 75^th^ percentiles, respectively 3.2, 4.5, and 7.6 μm. An additional group did not exhibit an undetectable lumen space. We also investigated the complexity basing on both the presence/absence of CD146^+^ perivascular cells, surrounding vessels, and tubular or no tubular appearance evidenced by Z-stack and 3D reconstruction (Figure 3B). A 5 × 3 contingency table was analyzed by the *chi square test*, with a resulting *p*-value lower than 0.0001 (Figure 3C). The column representing structures with CD146^+^ perivascular cells but not tubular appearance were not analyzed.

We found that 64 out of 487 NESTIN^+^ structures were not tubular or their lumen was not detectable, perhaps due to the limited section thickness (Figure 3B and Supplementary Video 1). These cells appeared to be single cells or small clusters (NCs) of NESTIN^+^ cells. Interestingly, all tubular structures (268 out of 487) that did not contain CD146^+^ pericytes (Figure 3B and Supplementary Video 2) exhibited an IC smaller than the 50^*th*^ percentile (4.5 μm). Most of these vessels showed imperceptible lumens (163 out of 487) and then classified as NCLTs. Conversely, most of the wider vessels (116 out of 155) showed sub-endothelial CD146-positive cells. EAs characterized by a single layer of perivascular cells, showed IC between 50^th^ and 75^th^ percentiles (4.5 and 7.6 μm, respectively), whilst wider arteries (As) were easily distinguishable based on their multilayered CD146-positive cells. It should be acknowledged that some structures (39 out of 155) exhibited an abnormal morphology due to unavoidable tissue compression during tissue processing and sectioning (white arrow in Figure 3A). These abnormally compressed vessels were named EAs of compressed lumens (cEAs, Figure 3C). In conclusion, about one in three NESTIN^+^ vessels was an artery (13.7%) or an endosteal arteriole (22.9%, including the cEA), whilst most NESTIN^+^ micro-vessels were NCLTs (63.4%, Figure 3D). Similar conclusions were obtained by detecting smooth muscle cells in arteries and arterioles with αSMA antibody (Supplementary Figure 5). The analyzed contingency table, with αSMA instead of CD146, resulted in a *p*-value lower than 0.005. Interestingly, no significant correlation has been detected among *NES*, *MCAM* (CD146) and *ACTA1* (αSMA) gene expression (Supplementary Data 2).

### Differential Expression of EMCN and vWF on NESTIN-Positive Vessels

**FIGURE 4:**
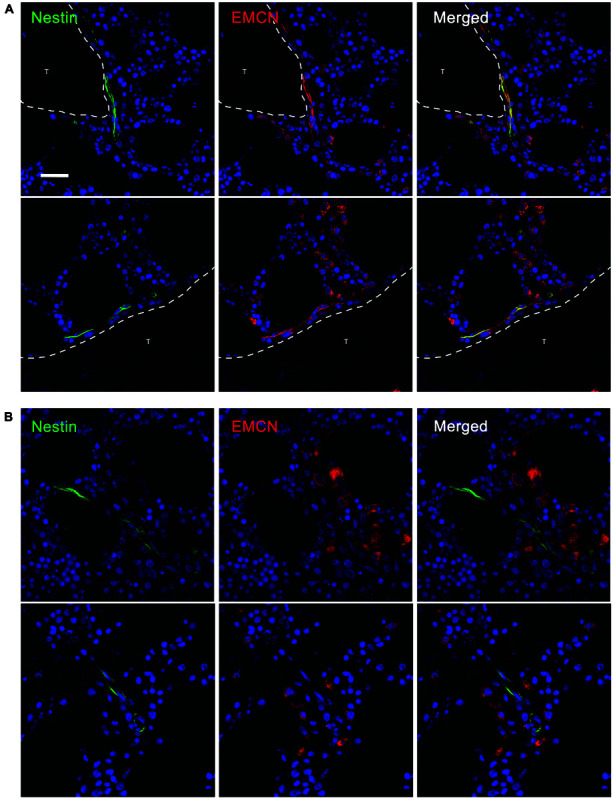
EMCN was detected only in NCLTs in close proximity of the bone line. **(A)** Most NESTIN (green)–positive NCLTs, detected in proximity of the bone line (<10 μm), showed positive stain for ENDOMUCIN (EMCN, red). **(B)** Conversely, when a bone trabecula was not detected in the FOV of NCLTs, this latest resulted negative for EMCN (T = bone trabecula, scale bar: 20 μm).

**FIGURE 5:**
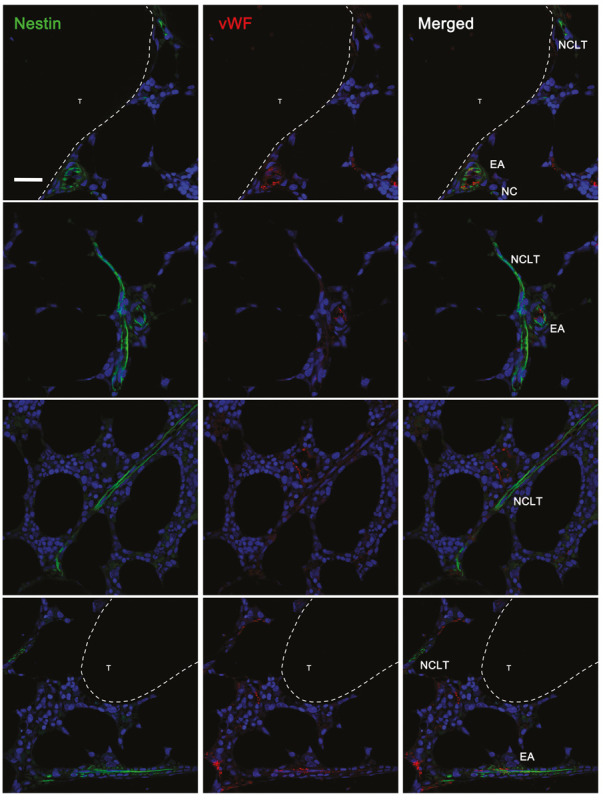
vWF was detected only in arterial and arteriolar BMECs not in NCLTs. von Willebrand factor (vWF) was detected in a fraction of NESTIN^*bright*^ (green) micro-vessels encircled by NESTIN^*dim*^ perivascular cells with elongated nuclei, in particular in endosteal arterioles (EA). All NCLTs resulted negative for vWF (T = bone trabecula, scale bar: 20 μm).

Interestingly, human A and EA aBMECs did not seem to express EMCN (data not shown), resembling the endothelium of larger “type L” vessels described, in mice, by Kusumbe et al. (2014) as distal arterioles. EMCN was instead expressed by 55% of the NCLTs, which shows great similarity with small “type H” micro-vessels previously described as capillaries, in the murine BM. Moreover, a significant correlation (*p* < 0.05) was detected between EMCN expression and distance from the endosteum. In fact, almost entirely of the EMCN-positive NCLTs were detected in close contact with a trabecula or bone-lining cells (Figure 4A), while none of the EMCN-negative NCLTs were in proximity of the bone line (Figure 4B). This is strongly reminiscent of high EMCN expression in transition zone vessels that support developmental bone growth (Ho et al., 2019). Conversely, a dishomogeneous expression of vWF was detected in some EAs and As, but not in NCLTs (Figure 5). These data are consistent with a positive correlation between *NES* and *EMCN* genes (*R* = 0.903, *p* < 0.05, Supplementary Figure 8A) in nucleated human BM cells.

### Endosteal Region and Close Spatial Correlation of NCLTs and Bone Trabecula

**FIGURE 6:**
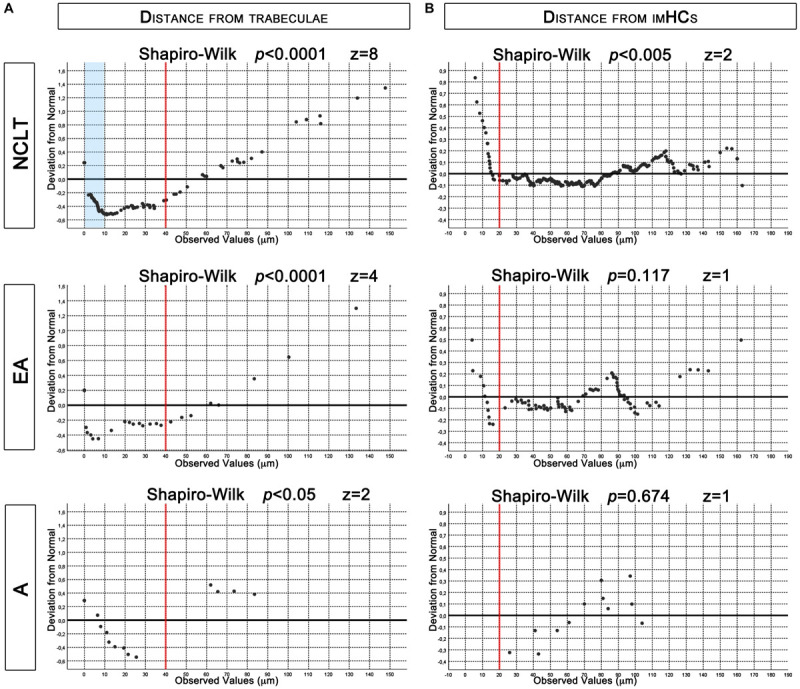
De-trended Q-Q plots of data distribution. **(A)** Asymmetrical distribution of measured distances from trabecula, reported for NCLTs and EA, revealed increased frequency of data between 0 and 40 μm (red lines). In this region data distribution of NCLT showed increased frequency of measures lower than 10 μm (pale blue box). Conversely in EA and A the data were symmetrically dispersed. **(B)** Even if potentially symmetric, measured distances from imHCs in NCLT resulted not normally distributed due to increased frequencies below 20 μm (red lines). Conversely, in EA and A the distribution resulted normal.

To evaluate the proximity of NESTIN^+^ structures to bone trabecula margins or to bone lining cells, we quantified their spatial relationships with the trabecula in the FOV and measured their overall distance from the bone surface. Almost half (45.7%) of the NCLTs microphotographs included bone in their FOV. This co-occurrence was significantly higher (*p* < 0.005, *n* = 268) than for the rest of NESTIN^+^ vessels. One third (31.6%) and one fourth (24.1%) of the EAs and As microphotographs, respectively, included bone areas. Only arteries reported a statistical significance (*p* < 0.01, *n* = 58) of the reduced frequency respect to the rest of the NESTIN^+^ micro-vessels. The analysis of the distribution of the measured overall distances revealed an asymmetric right/positive-skewed distribution both for NTLCs (Shapiro–Wilk *p* < 0.0001, skewness = 1.734 *z* = 8, *n* = 123) and EAs (Shapiro–Wilk *p* < 0.0001, skewness = 1.539 *z* = 4, *n* = 31). Most of the data reported showed distances between 0 and 40 μm (left of the red line in Figure 6A). By contrast, a very low number of trabecula in microphotographs of arteries did not allow a correct evaluation of the data distribution, which however appeared potentially normal (Shapiro–Wilk *p* < 0.05, skewness = 0.939, *z* = 2, *n* = 14). Interestingly, analyzing the distribution of data between 0 and 40 μm a systematic deviation from normality (Shapiro–Wilk *p* < 0.0001, skewness = 1.095, *z* = 4, *n* = 96) was evident in the NCLTs series for data reporting distances shorter than 10 μm (pale blue area in Figure 6A). Conversely, data distribution of distances in the range of 0–40 μm around EAs (Shapiro–Wilk *p* < 0.0001, skewness = 0.715, *z* = 1, *n* = 23) and As (Shapiro–Wilk *p* = 0.844, skewness = 0.098, *z* = 0, *n* = 10) resulted potentially symmetric (Figure 6A). A portion of trabecula was frequently detected in the FOV of NCs (43.7%) and most frequently within 40 μm from the bone line (Shapiro–Wilk *p* < 0.0001, skewness = 1.309, *z* = 3, data not showed). Taken together these results suggest that NESTIN^+^ EAs and NCLTs are located in the endosteal BM region (conventionally defined within 40 μm from the bone surface).

### Spatial Correlation of NCLTs and Non-endothelial CD34^+^ Cells

Three-color immunofluorescence image analysis allowed us to readily detect single and rounded CD34^+^ showing high N/C ratio and to discriminate them from CD34^+^ BMECs because of their characteristic dotted pattern (red in Supplementary Figure 1, upper panels; yellow in Supplementary Figures 6, 7). This CD34-positive cell population could not be further characterized due to the lack of high-quality monoclonal antibodies, against CD90 or CD38 for instance, suitable for confocal imaging and produced nor in mice or rabbits. However, the human BM CD34^+^ cells are mostly immature hematopoietic cells (imHCs) (Pellin et al., 2019) and independently by their differentiating stage, are here indicated as imHCs. The occurrence of at least a single imHCs in the FOV resulted higher than 90% in the NCLTs (95.6%), EAs (91.3%), and NCs (90.9%) series, supporting the representativeness of the areas sampling. Conversely, only 50.0% of As microphotographs contained imHCs.

The analysis of the distribution of the overall distances of the imHCs detected in NTCLs series, revealed a potentially symmetrical (but not normal) distribution (Shapiro–Wilk *p* < 0.001, skewness = 0.274, *z* = 2, *n* = 300). Analyzing the de-trended Q-Q plot the deviation from normality should be ascribed to a consistent increase in the frequency of data shorter than 20 μm (left of the red line in Figure 6B). Dissecting the data series, analysis of distribution resulted normal within 20 μm (Shapiro–Wilk *p* < 0.0001, skewness = 0.435, *z* = 3, *n* = 25) and positively skewed (Shapiro–Wilk *p* = 0.674, skewness = 0.321, *z* = 1, *n* = 275) for values >20 μm. The results strongly support a spatial correlation of NCLTs and CD34^+^ imHCs located within a perivascular region of 20 μm from these NESTIN-positive capillaries. Conversely, EAs (Shapiro–Wilk *p* = 0.674, skewness = 0.321, *z* = 1, *n* = 79) and As series resulted normally distributed (Shapiro–Wilk *p* = 0.673, skewness = −0.319, *z* = 1, *n* = 12). Of note, no imHCs were detected within 20 μm from As (Figure 6B).

## Discussion

In the present paper, we performed extensive image analysis to characterize NESTIN expression in aBMECs and perivascular cells from human adult benign biopsies. The positive stain for NESTIN revealed very small capillary-like tubular structures here named NESTIN-positive capillary tubes (NCLTs), spatially associated with the bone surface and imHCs and in addition to arteries (As) and EAs previously described in mice (Itkin et al., 2016). We found high levels of NESTIN in CD34^+^CD31^+^ arterial/arteriolar BMECs and comparatively lower but positive NESTIN expression in the perivascular region. Sinusoids were NESTIN^*neg*^ both in BMECs and their perivascular cells.

Our data, regarding the co-localization of human NESTIN with CD34 and CD31 are consistent with previous studies in mice indicating the presence of NESTIN^+^ cells in arteries and transition zone vessels associated with bone (Itkin et al., 2016; Ho et al., 2019). Although the original isolation method excluded CD31^+^ NESTIN^+^ cells (Méndez-Ferrer et al., 2015), subsequent studies have demonstrated that both in mouse and human, NESTIN^+^ endothelial and perivascular cells are found in arteriolar vessels. Of interest, Isern et al. (2014) detected CD31^+^ endothelial cells within the *Nestin-gfp*^+^ population in fetal BM and showed their number to increase during development. Ono et al. (2014) demonstrated that endosteal osteoblasts, perivascular stromal as well as endothelial cells express NESTIN in developing murine BM. Authors reported differences in the cell types tagged applying Nes-GFP or Nes-creER, showing that Nes-creER preferentially targets an endothelial subpopulation of NESTIN^+^ cells. Interestingly, about 20% of Nes-creER cells were Cxcl12-GFP+ arterial/arteriolar BMECs, proposing that NESTIN^+^ cells could have an HSPC-supporting activity. Later, The presence of NESTIN^+^ cells within the endothelial CD31^+^ fraction was confirmed also in adult mice (Itkin et al., 2016).

Our results are in accordance with the scarce evidence available for human NESTIN^+^ aBMECs. Indeed, the presence of bright NESTIN stain in CD34^+^ CD31^+^ endothelial cells of capillaries/arterioles in human benign, leukemic and myelodysplastic bone biopsies has been recently reported, whereas sinusoids appeared negative or weakly positive (Flores-Figueroa et al., 2012). Moreover, these authors found half of CD34^+^ HSPCs within 10 μm of distance from unspecified vessels, both in benign and dysplastic human BM TMA. We report here a similar spatial association with NCLTs, which comprise more than 60% (268) of the total analyzed BM vessels (423).

This abundance of NCLTs could be interpreted as a cutting plane effect due to the high complexity of a tangled capillary network, that we described contiguous with the endosteum. As a consequence, the high frequencies of CD34^+^ cells around NCLTs would imply proximity of imHCs to the bone-lining cells. Thus, the idea of disconnected “perivascular” and “endosteal” hematopoietic niches supporting different HSPC fates should be re-discussed as already suggested by Cordeiro-Spinetti et al. (2015), particularly considering that the “perivascular niche” was usually identified in the perisinusoidal region. Indeed, a different involvement of periarteriolar and perisinusoidal regions has been recently suggested (Morrison and Scadden, 2014), and recent work by Xu et al. (2018) supports the hypothesis of a specific arteriolar microenvironment for distinct HSPC subsets, sustained by the selective expression of niche factors.

A major novelty aspect of this study is the identification of NCLTs in the human BM, probably part of the transitional zone capillary network, resembling previous findings by Li et al. (2009) in mice. NCLTs form an endosteal capillary network that seems to connect A/EA with S near the bone (Supplementary Figure 9). Brookes and Revell (1998) first described “transition points” between arterioles and sinusoids called “arterio-venous junctions.” Further studies in Tie-2-gfp transgenic mice injected with Dil-Ac-LDL (Li et al., 2009) allowed to better characterizing the architecture of transitional vessels. Arteriolar BMECs showed evident GFP expression throughout cells and were unable to internalize Dil-Ac-LDL while sinusoidal BMECs appeared GFP^*neg*^ with intense Dil-Ac-LDL uptake. GFP expression was detected also in straight capillaries (4–6 μm in diameter) that closely resemble the human NCLTs we described here. The straight capillaries connected with larger vessels (10–15 μm), characterized by perinuclear residual GFP expression and Dil-Ac-LDL endothelial endocytosis that emptied into sinusoids. Analyzing the microanatomy of the transition zone, the straight capillary network occupied a small percentage of the area and showed higher density in the proximity of the endosteal surface (Li et al., 2009; Ho et al., 2019). This is in accordance to what we report here for the endosteal capillary network in humans suggesting that NCLTs could act as transitional capillaries.

Itkin et al. (2016) proposed a different picture of the transition zone in mice, suggesting an arteriole-sinusoid interphase but devoid of capillaries clearly distinguished from arterioles. Here we present an extended and more detailed classification of BM micro-vessels in humans, which shows a remarkable similarity with the mouse BM. Using CD34 instead of Sca-1, we confirmed the distinction between NESTIN^*bright*^ arteries/arterioles and NESTIN^*neg*^ sinusoids. We also confirmed the morphometric distinction between arteries (A) and EA proposed by Itkin’s group that reported, respectively >10 μm and 5–10 μm as diameter ranges, very close to the cut-offs of >7.6 μm for As and 4.5–7.6 μm for EAs, here reported in humans. According to Itkin’s description in mice, approaching the endosteum (<40 μm) arteries (A) branched into the smaller arterioles (EA) that were not associated with αSMA^+^ pericytes, but were instead surrounded by NESTIN^*dim*^ mesenchymal cells and clusters of HSPCs. This is in great accordance with our data taking into account that in humans, NESTIN^*dim*^ MSC has been reported expressing both αSMA and CD146 (Corselli et al., 2013). Moreover, our results are partially in accordance with the BM taxonomy recently proposed by Baryawno et al. (2019) in mice, indicating the characteristic expression of CD34 in aBMECs and vWF expression distinguishing arterial from arteriolar vessels. However, in this RNAseq paper arterioles (supposedly similar to EAs) could not be discriminated from capillaries (NCLTs) in the absence of a morphometric study or specific markers. Thus, it is reasonable to hypothesize that some vWF-negative cells classified by Baryawno et al. (2019) as aBMECs were instead capillary BMECs (cBMECs), similar to those constituting human NCLTs. Of note, our human study is also consistent with the finding by Baryawno et al. (2019) of EMCN distinguishing endosteal from non-endosteal mouse BM vessels, supporting our data in human NCLTs.

Considering their micro-anatomic localization, it might be reasonable hypothesize that NCLTs may be involved in trabecular bone remodeling. Studies on rat model showed that bone formation rates correlate with capillary density in BM and that PTH spatially relocates blood vessels closer to sites of new bone formation (Prisby et al., 2011). In human adults, Andersen et al. (2009, 2013, 2014) investigated the pathophysiological mechanism coupling bone resorption and formation, describing specialized structures named “canopies” that cover the entire remodeling site and separate it from the marrow cavity. Of note, these canopies have been described being rich in capillaries, and pre-osteoclasts have been described localizing preferentially along them. Similarly, applying biopsies from iliac crest of human donors, Kristensen et al. (2013) reported an increased capillary density next to the remodeling sites in cancellous bone coupled with a significantly increased number of putative osteoblast progenitors and proliferative cells in a region within 50 μm from the bone line or the canopy surface, and even higher when in close contact with them. Furthermore, the increased number of capillaries detected during bone remodeling has been reported being specifically associated to the increased number of CD31^*high*^EMCN^*high*^ type H vessels (Kusumbe et al., 2014; Peng et al., 2020), which resemble the NCLTs as discussed above, strengthening the hypothesis of the NCLT involvement in the trabecular bone remodeling process.

In line with this hypothesis, we described here single or small clusters of CD34^+^ NCs located in the endosteal region. The anatomic co-localization and relative proximity suggest a possible relationship between NCs and NCLTs, because NCs might represent progenitor cells that retain the potential to generate NCLTs, mimicking the role of Endothelial Progenitor Cells (EPCs) in neovascularization (Gao et al., 2009; Li et al., 2012). Suzuki et al. (2010) reported significant expression of NESTIN in BM-derived EPCs and proliferating endothelial cells in mice. In this scenario, endosteal NESTIN^+^ capillary network could be involved during the physiological remodeling of the endosteum in post-natal BM, and that the new endosteal capillaries formation could be also supported by the vasculogenesis starting from NCs rather than sprouting or intussusception angiogenesis from pre-existing NCLTs (Ishige-Wada et al., 2016) or possibly by a combination of the two.

Despite recent advances in *in vivo* imaging and genetic tracing, relevant issues on the biology of HSPC niches remain open (Ishige-Wada et al., 2016; Crane et al., 2017). In particular, the use of animal models as predictive tools for human BM requires additional human studies addressing interspecies similarities and differences. A different spatial arrangement of capillaries and sinuses networks in human iliac crest respect to the mouse femora has been hypothesized (Chen et al., 2017). This suggests that, together with the plausible inter-species variability, the different ossification pathway could be relevant (Steiniger et al., 2016). Steiniger et al. (2016) studied the arrangement of human BM micro-vessels applying 21 serial sections of about 1 cm^2^ derived from a single human iliac crest specimen. In this work, the distinction between sinusoids and capillaries was based on the inner caliber and expression of CD141 and CD34, where small CD34^+^CD141^*neg*^ vessels were described as capillaries; consequently, perivascular cells were not investigated. In addition, micro-vessels smaller than 7 μm were removed during the rendering of 3D models and excluded in this study. Flores-Figueroa et al. (2012) described small NESTIN^+^CD34^+^ arterioles enwrapped by CD146^+^αSMA^+^ vascular smooth muscle/pericyte (VSMP) layer, in accordance with our data. Moreover, these NESTIN^+^ CD34^+^ arterioles have been reported in association with arborizing CD271^+^ MSC-like cells but separated from them by the continuous VSMP layer. Conversely, NESTIN^*neg*^ sinusoidal endothelium, lacking in the VSMP wrapping, has been reported in direct contact with the CD271^+^ MSCs. These data confirmed previous observations in human BM trephines from Tormin et al. (2011) that reported bright CD146^+^αSMA^+^ cells within the *tunica media* of small and larger arteries, whereas CD271 expression resulted detected exclusively in the *tunica adventitia* by αSMA^*dim*^ cells or in the perisinusoidal region. Thus, the detection of bright CD146^+^αSMA^+^ VSMP layer seems a more stringent criterion to dissect arteries and arterioles from capillaries and sinusoids, compared with CD271^+^. Among BM vessels that lack CD146^+^αSMA^+^ VSMP layer, NCLTs could not be confused with sinusoids taking into consideration their inner caliber and CD34/CD31 expression. Ewalt and Gratzinger (2014) already suggested a more stringent phenotype to discriminate NESTIN^*bright*^CD31^*bright*^CD34^*bright*^CD105^*dim*^ arterioles and capillaries from NESTIN^*dim/neg*^CD31^*dim*^CD34^*dim*^CD105^*bright*^ sinusoids, albeit without clarifying the distinction between capillaries and arterioles. In the present paper, we provide novel insights and partially confirm these phenotypes for arteriolar and capillary BMECs (cBMECs) as clearly distinct from CD31^*neg*^CD34^*neg*^ sinusoidal BMECs. Here we propose a characterization based on stringent criteria to dissect As, EA, and NCLT among CD34^+^ micro-vessels. Moreover, we believe micro-anatomic localization to be crucial to characterize the role of different arterial and capillary BMEC micro-vascular networks. Our data showing that this endosteal capillary network is localized within 40 μm from the bone line and getting in touch with it by cBMEC in direct contact with the bone-lining cells. Moreover, the reported spatial association of HPSCs with NCLTs could be suggestive for a specialized hematopoietic microenvironment in this region. Even if far from demonstrating a possible “*endosteal capillary niche*,” our results showed that imHCs are not randomly distributed in this area but significantly condensed within 20 μm from the NCLTs.

A number of groups have already performed similar analyses in the murine model to demonstrate a spatial correlation between HSPCs and the microvasculature. In these papers, spatial statistical methods known as points processes have been applied using randomly generated spots, to be compared to the HSPCs distribution (Acar et al., 2015; Gomariz et al., 2018). This complex 3D mathematical modeling of the BM cavity requires the confocal imaging reconstruction of the whole bone (usually femurs or sternum) and algorithms taking into account the heterogeneity of this tissue. Because this method cannot be applied to human BM trephines, we have used an alternative approach allowed applying data distribution analysis, whereby the observed values were compared to the Gaussian curve that represents a random distribution.

In conclusion, the extensive image analysis allowed us to dissect the NCLT vascular network, whose characterization in mice and humans has been largely neglected thus far probably due to the very small IC of these vessels. It is reasonable to hypothesize that the endosteal capillary network could be specifically involved during blood vessel re-modeling, as previously demonstrated in acute myeloid leukemia (AML) models by using the intra-vital imaging. In particular, endosteal CD31^+^ENDOMUCIN^+^ small vessels were decreased respect to the central BM microvasculature (Duarte et al., 2018; Ho et al., 2019). In this regard, Baryawno et al. (2019) reported an increased number of arteriolar/capillary BMECs detected by scRNA-seq in a mouse AML model. Altogether, these findings suggest destruction of the endosteal niche in favor of a possible increase of AML supporting vasculature in central BM. However, further studies are required to clarify the endosteal capillary plexus role as HSPC niche, in BM re-modeling in homeostasis and pathological disorders. Nonetheless, our findings highlight the importance of BM microvasculature characterization at a cell resolution level that allows the dissection of arteriolar from the capillary network.

## Data Availability Statement

All data generated or analyzed during this study are included the article/Supplementary Material.

## Ethics Statement

This study has been performed according to the declaration of Helsinki. No informed consent was required for anonymized archival bone marrow biopsies. Paraffin blocks were stored in the archives of the Pathology department of “Santa Chiara” University Hospital in Pisa and serve as legal proof of previous diagnostic investigations. No samples were obtained directly from patients, thus the research presents less than minimal risk of harm to subjects, and the waiver of consent did not adversely affect the rights and welfare of subjects. The research did not use a tissue block in presence of the slightest suspicion that this could hinder future clinical use such as re-evaluation or further studies. The study received the approval from the ethics committee of the “Azienda Ospedaliero-Universitaria Pisana – Comitato Etico di Area Vasta Nord Ovest (CEAVNO)” (committee approval number: 10148/20) that also waived the need for informed consent for the study.

## Author Contributions

FP and SP were responsible for conception and design, acquisition, analysis, interpretation of data, and drafting the manuscript. MM, SB, and SM were responsible for the acquisition of data. RM was responsible for statistics. EC was responsible for the selection of the samples. VC was responsible for qPCR data acquisition. MP was responsible for revising the manuscript critically for important intellectual content and final approval of the version to be published. All authors read and approved the final manuscript.

## Conflict of Interest

The authors declare that the research was conducted in the absence of any commercial or financial relationships that could be construed as a potential conflict of interest.
