# Supplementary material for: High NESTIN Expression Marks the Endosteal Capillary Network in Human Bone Marrow
Source: Front Cell Dev Biol. 2020 Dec 8;8:596452. doi: 10.3389/fcell.2020.596452 (PMC7753038; doi:10.3389/fcell.2020.596452)
Supplement: Supplementary file 1 [file Data_Sheet_1.PDF]

Supplementary Material

1 Detailed Materials and Methods

1.2 Immunohistochemistry and Immunofluorescence

Slides were obtained from de-identified archival paraffin-embedded BM biopsies showing normal histology, hereinafter refer as “benign biopsies”. Bone marrow trephines were obtained from iliac crest of 17 patients (11 male and 6 female, median age: 57), 13 staged for lymphoma (2 Hodgkin’s and 11 non-Hodgkin’s) and four evaluated for cytopenia. Sections of 3 μm were prepared by Leica RM 2155 microtome (Leica Biosystems, Wetzlar, Germany) and gently layered on Polysine® Slides (Thermo Scientific, Waltham MA, USA). Sections were deparaffinized in Bio-Clear (Bio-Optica, Milan, Italy) and hydrated by graded alcohol series.

Antigen microwave retrieval was performed in citrate buffer (100 mM), pH 6.0. Immunohistochemistry was carried out using mouse monoclonal anti-human NESTIN (1:150, clone 10C2, Abcam, Cambridge, UK) by SuperPicture™ 3rd Gen IHC Detection Kit (Thermo Scientific), according to manufacturer’s. Primary antibody incubation was performed overnight at 4 °C. Sections were then counterstained with Gill’s n.3 hematoxylin (Sigma-Aldrich, Saint Luis MO, USA). Proper mouse isotype control (Abcam) was used as negative control. Immunohistochemistry imaging was carried out using DMR Leica microscope equipped with LAS v 4.4 Software (Leica Biosystems).

In parallel, re-hydrated slides were permeabilized in 0.5% Triton X-100 for 15 min and reaction blocked by Image-iT™ FX signal enhancer (Thermo Scientific) for 30 min. Primary antibody staining was performed overnight at 4 °C using mouse monoclonal anti-human NESTIN (1:150); rabbit monoclonal anti-human CD146 (1:200); rabbit polyclonal anti-human CD34 (1:150), anti-human alpha Smooth Muscle Actin (α-SMA, 1:200), anti-human ENDOMUCIN (EMCN, 1:200), anti-human CD31 (1:50) and anti-human von Willebrand factor (vWF, 1:500) antibodies (Abcam, Cambridge, UK). Immunofluorescence was carried out by goat anti-mouse SFX kit (Thermo Scientific), according to manufacturer’s using AlexaFluor®-488 anti-mouse IgG and AlexaFluor®-555 anti-rabbit IgG (Thermo Scientific). Appropriate isotype controls were performed in parallel for each primary antibodies panel applied. Slides were mounted in ProLong™ Diamond anti-fade reagent with 4′,6-diamidino-2-phenylindole (DAPI, Thermo Scientific) for nuclei detection. Immunofluorescence imaging was performed by SP8 Confocal Microscope equipped with LAS X Software (Leica Biosystems) and 3D reconstructions were obtained after Z-stack acquisition (steps of 0.35 μm).

1.3 Image Analysis

Immunofluorescence photomicrographs (no less than 10/sample) were taken at 63x, with camera field of view (FOV) covered by the tissue images (FOV= 184,52 μm x 184,52 μm). Pictures showing NESTIN^{+} structures were included in the study and processed to measure vessel inner calibre (IC). In transversal sections IC measures were taken along the minor axis, in longitudinal sections the widest visible calibre was deemed as IC (Supplemental Figure S1). The presence of CD146^{+} perivascular cells was recorded and CD34^{+} cells were counted. Distance of CD34^{+} cells from vessels was measured from the cell nucleus to the midpoint of IC segments (Supplemental Figure S1, upper panels). Distances were normalized by subtracting half of the IC. Vessel distance

from trabeculae was obtained by tracing the measuring line perpendicular to “bone line”. “Bone line” was detected during scanning of the entire trephine slice area in epifluorescence in order to center a NESTIN-positive structure in the FOV. During FOV selection in epifluorescence the presence of mineralized area was evident thank to the natural auto-fluorescence of the *trabeculae* that showed typical lamellar pattering and sporadic embedded cells (osteocytes, revealed by DAPI). If a *trabecula* was included in the FOV, the green epifluorescent image was taken before switching to the confocal mode. The epi-green picture was then temporarily stacked with TOPO-3 (nuclei stain) acquired channel and the “bone-lines” were then manually marked to allow measuring distances in the final merged image (Supplemental Figure S1, lower panels). All measures were obtained applied LAS X Image Analysis Software (Leica Biosystems).

1.4 *Co-localization analysis*

Two-color channel images were obtained splitting channels by NESTIN/CD34//DAPI and NESTIN/CD31/DAPI microphotographs. NESTIN (Channel 1) and CD34/CD31 (Channel 2) were then converted to gray-scale by removing pseudocolors and processed for rolling ball background subtraction with radius of 50.0 pixels, applying ImageJ Software (National Institute of Health, Bethesda MD, USA). Co-localizzation analysis was performed by the “*Coloc 2*” Fiji’s plugin for ImageJ, applying Costes’ auto-threshold after 100 cycles of randomization [33], Pearson’s and Mander’s coefficient values were recorder for pixels above the threshold. Co-localization negative control images were obtained translating the “Channel 1” with 100 pixels offsets in both on X- and Y-axis.

1.5 *Four-color immunofluorescence using specific CD34 dual color co-labeling*

Slides were incubated with primary antibody overnight at 4 °C, using mouse monoclonal anti-human NESTIN (1:150) and rabbit polyclonal anti-human CD34 (1:150). After three washes (5 min each), secondary antibody incubation with AlexaFluor®-647 anti-mouse IgG and AlexaFluor®-555 anti-rabbit IgG (Thermo Scientific) was performed (1 h). After extensive washing, primary CD146 (rabbit polyclonal anti-human) antibody was applied for 1h at 4 °C. Slides were washed 3 times and incubated with AlexaFluor®-488 anti-rabbit IgG for 1h. Pictures were captured using SP8 Confocal microscope. Blue pseudocolor was associated to AlexaFluor®-647 emission (NESTIN), red to AlexaFluor®-555, green to AlexaFluor®-488, and cyan to DAPI. $CD34^{+}$ cells were evidenced by a yellow pseudocolor as overlap of the green and red channels due to the co-labeling of AlexaFluor®-555 and AlexaFluor®-488 secondary antibodies (Supplemental Figure S2). In parallel, rabbit monoclonal IgG isotype control (Abcam) was applied in place of anti-CD34 and CD146 antibodies.

1.6 *Statistics*

Histomorphometric data regarding inner calibre, presence of perivascular cells and tubular shape were analyzed in a 5x3 contingency table. The dichotomous values “presence/absence of perivascular cells” and “tubular/no tubular shape” were combined with the calibre measures divided into quartiles, *chi-square* test was applied. The distribution of data regarding distance from bone line and HSPCs were evaluated by graphical method (Q-Q plot chart) and normality was analyzed by Shapiro-Wilk test. Comparisons between of co-localization mean coefficients were performed applying Kruskal-Wallis ANOVA test and Dunns’ post-test. All analyses, descriptive and inferential, were carried out by SPSS v.25 technology.

2 *Supplementary Data*

2.1 MATERIALS & METHODS

Human BM aspirates were collected in K_3EDTA pre-loaded Vacutainer® tubes (Becton Dickinson, Franklin Lakes, USA-NJ) by iliac crest puncture from four patients (3M, 1F median age 53) staged for non-Hodgkin's lymphoma. After the diagnostic investigations, discarded excess blood samples were de-labeled and sent to our laboratories. The study was performed according to the declaration of Helsinki and sample collection protocol approved by the ethical committee of the "*Azienda Ospedaliero-Universitaria Pisana - Comitato Etico di Area Vasta Nord Ovest (CEAVNO)*" (committee approval number: 10148/20). Red blood cells were removed from samples by 15' incubation with Red Blood Cell Lysis Solution from Miltenyi Biotec (Bergisch Gladbach, Germany), at room temperature. Samples were centrifuged at 400 g for 5 min and BM cells were counted by Bürker hemocytometer.

2.1.1 *Flow cytometry*

BM-derived cells were processed for multicolor flow cytometry incubating for 30 min at 4°C with fluorochrome-conjugated antibodies: anti-CD34 VioBlue®, anti-CD31 PE-Cy7 (Miltenyi Biotec). After washing in MACSQuant® Running Buffer (Miltenyi Biotec), cells were fixed and permeabilized applying Inside Stain kit (Miltenyi Biotec) for intra-cellular detection of NESTIN by incubation with mouse monoclonal anti human-NESTIN (1:100, clone 10C2, Abcam, Cambridge, UK) for 1 h at 4°C. After washing in permeabilizing solution, NESTIN was revealed by AlexaFluor®-488 conjugated anti-mouse IgG (Thermo Fisher) for 1h at 4°C. Cells were then washed twice and at least 100'000 events were acquired by MACSQuant® Analyzer (Miltenyi Biotec) equipped with MACSQuantify® analysis software (Miltenyi Biotec). After exclusion of debris on SSC *vs* FSC plot, doublets on FSC-H *vs* FSC-A and dead cells on SSC-A *vs* 7-AAD, NESTIN-positive events were gated on SSC-A *vs* B1-A density plot and displayed on V1-A (CD34) *vs* B4-A (CD31) dot plot. IgG isotype control was also performed to subtract percentage of non-specific signals.

2.1.2 *Human BM-derived cell fractioning*

Human BM-derived cells were processed for immunomagnetic isolation applying anti- human CD105 MicroBeads Kit (Miltenyi Biotec) and AutoMACS® Pro Separator (Miltenyi Biotec), according to manufacturer's instructions. Positive and negative fractions were then collected after running sensitive positive selection program, and immediately processed for mRNA extraction. CD105 negative fraction were then incubated with anti- human CD31 MicroBeads and processed to collect $CD105^{neg}CD31^{+}$ and $CD105^{neg}CD31^{neg}$ cell population.

2.1.3 *Gene expression analysis.*

Total RNAs were purified from freshly sorted cells by Direct-zol RNA MicroPrep Kit (Zymo Research, Irvine, USA-CA) and quantified with Qubit 4 Fluorometer (Thermo Fisher, Waltham, MA-USA) by Qubit RNA HS Assay Kit (Termo Scientific). cDNAs were synthesized from 1 µg total RNA using iScript gDNA Clear cDNA Synthesis Kit, according to manufacturer. qPCR was carried out by SsoAdvanced Unversal SybrGreen Supermix (BioRad) on iQ5 Real time PCR Detection System (BioRad), according to PrimePCR Array™ manufacturer. *NES* and *PECAM1* gene expression was evaluated on immunomagnetic sorted cells using specific custom designed primer pairs (*NES* sense: GTTGGAACAGAGGTTGGAG; *NES* anti-sense: GAGGGAAGTCTTGGAGCC *PECAM1* sense: GAACCTGTCCTGCTCCATC; *PECAM1* anti-sense: TCAAACTGGGCATCATAAGAAAT. Fold change $\Delta\Delta C_t$ method calculations and statistical

analysis were carried out using PrimePCR™ Analysis software (BioRad). C_t values over 35 were considered as "no expression". Following best housekeeping gene test, *HPRT1* and *GAPDH* were selected for normalization.

Whole white bone marrow cells were instead assayed applying custom designed 96-well PrimePCR™ Plates (BioRad, Hercules, USA-CA) containing primer sets for 86 target genes of interest, 5 reference genes (*ACTB*, *B2M*, *GAPDH*, *HPRT1*, *RPL13*) and 5 internal controls, used for gene expression profiling in a previous paper [61]. List of assayed genes are available as supplemental material of reference #57 and including, in addiction to *NES*, vascular endothelial markers (*CD34*, *PECAM1*, *KDR*, *EMCN*, *TEK*, *TIE1*, *FLT4*, *vWF, DLL4, LDLR, TEM1*), pericyte markers (*MCAM*, *ACTA1, RGS5*, *CSPG4*), MSC markers (*LEPR*, *NT5E*, *DES*, *RUNX2*) terminal differentiation-related mesenchymal markers (SPP1, SOX9, PPARG) and also lymphatic endothelial markers (*PDPN*, *LYVE1*, *PROX1*). Un-normalized ΔC_t expression of *NES* was correlated with expression of all other genes of interest and Pearson's test was performed applying GraphPad Prism® software (GraphPad Software, San Diego, USA-CA). C_t values over 35 were considered as "no expression".

2.2 RESULTS

Analysis of correlation of NESTIN gene expression and vascular endothelial markers revealed significant positive correlation with *PECAM1* (CD31, R= 0.907, p<0.05) and *CD34* (R= 0.905, p<0.05) corroborating the immunofluorescence evidences that NESTIN is expressed on endothelial cells. In addiction, *EMCN* and *TIE1* also significantly correlate with the *NES* expression (respectively R= 0.903, p<0.05 and R= 0.905, p<0.05). All other genes of interest, as *CDH5* (VE-Cadherin, *KDR* (VGFR-2) and *TEK* (Angiopoietin-1 receptor), did not report any significant correlation and *vWF* was not amplifiable. Interestingly, *FLT4* (VEGFR-3) negatively correlate with *NES* (R= -0.962, p<0.05, Supplemental Figure S8 A). These data are congruent with the immunological characterization of NESTIN-positive vascular structures in bone marrow biopsies proposed and with the literature, where NESTIN resulted highly expressed on VE-cadherin$^+$CD31$^+$CD34$^+$VEGFR-3^{neg} arteriolar and capillary bone marrow endothelial cells [62]. No significant correlation has been detected among *NES* and any pericytes markers, this is possibly due to the very low level of NESTIN expression detected in these cells by both qPCR and immunofluorescence. Conversely, *ALCAM* (CD166) and *NT5E* (CD73) MSC markers positively correlated with *NES* expression (respectively, R= 0.925, p<0.05 and R= 0.905, p<0.05, Supplemental Figure S8 B). *NES* expression on MSCs has been also confirmed applying more sensitive qPCR assay on fractionated cell populations able to detect *NES* in mesenchymal CD105$^+$ fraction even if 3 log lower respect to CD105^{neg}CD31$^+$ (p<0.001). Non-endothelial/non-mesenchymal CD105^{neg}CD31^{neg} population did not express *NES* (Supplemental Figure S8 C).

Flow cytometry revealed that most (71.5%) of the rare bone marrow NESTIN-positive cells (0.46±0.12% of total nucleated cells) resulted CD31^{bright}CD34$^+$ (Supplemental Figure S8 D), further confirming the preferential endothelial localization of NESTIN.

2.3 REFERENCES

61. Pacini S, Montali M, Mazziotta F, *et al*. Mesangiogenic progenitor cells are forced toward the angiogenic fate, in multiple myeloma. *Oncotarget*. 2019;10(63):6781-6790. doi: 10.18632/oncotarget.27285

62. Ramalingam P, Poulos MG, Butler JM. Regulation of the hematopoietic stem cell lifecycle by the endothelial niche. *Curr Opin Hematol*. 2017;24(4):289-299. doi: 10.1097/MOH.0000000000000350

3 *Supplementary Figures*

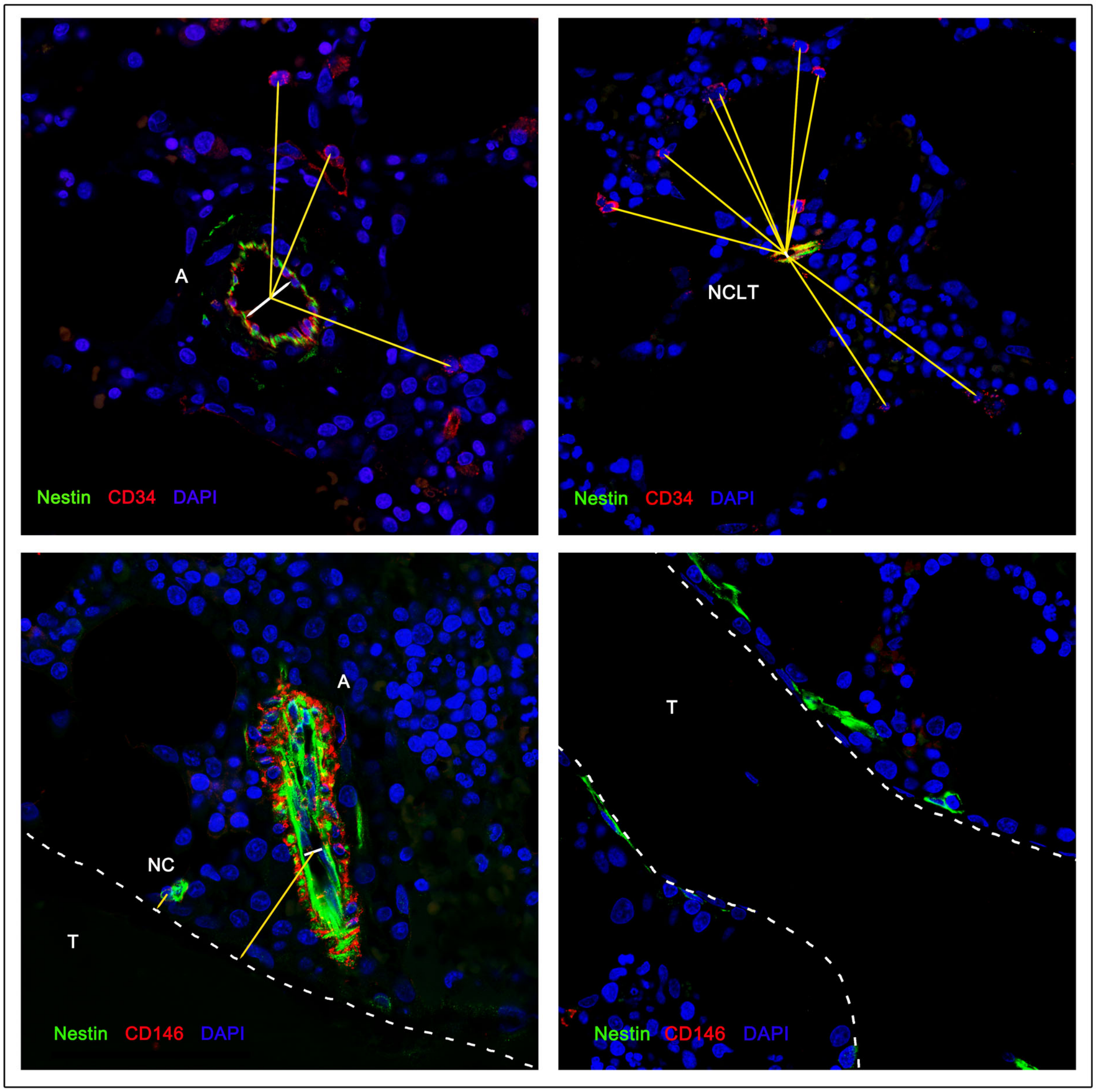

Supplemental Figure S1. *Criteria for vessel inner calibre (IC) and distance measurements*. Inner calibres were measured along vessel minor axis in transversal sections (upper left panel) or in correspondence of the wider visible lumen for longitudinal sections (lower left panel). HSPC distances (yellow segments in upper panels) were measured from the center of micro-vessels (mid-point of segments used for IC measurement: white lines) to the center of cell (dotted red in upper panels) nuclei. Distances from bone trabeculae were measured from the center of the micro-vessels to the trabeculae, along segments perpendicular to the bone line. NC distances were measured from the center of the cell nucleus closest to bone trabeculae (lower left panel). In the lower right panel we show an example of not quantifiable distance from trabeculae, referred to as direct contact.

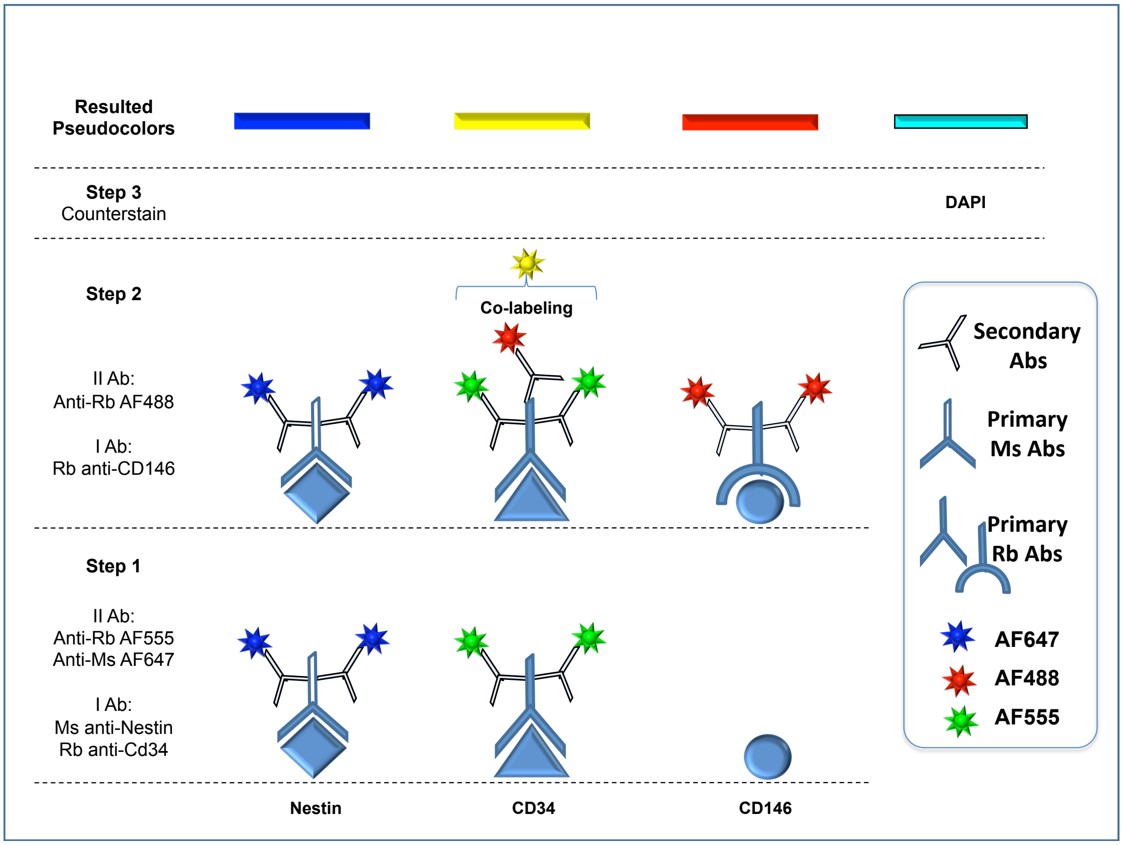

Supplemental Figure S2. *Forced co-labeling strategy*. Four-colour specific immunostaining was performed using mouse anti-NESTIN monoclonal and rabbit anti-CD34/CD146 polyclonal antibodies. Slides were incubated with anti-NESTIN and anti-CD34 followed by AlexaFluor®-647 and AlexaFluor®-555 to reveal the blue and green pseudo-colours, respectively. After extensive washing, slides were incubated with anti-CD146 followed by AlexaFluor®-488 to reveal the red pseudo-colour. In $CD34^+$ cells AlexaFluor®-488 and AlexaFluor®-555 are forced in a co-labeling reaction that originates the yellow pseudo-colour. DAPI signal was displayed in cyan.

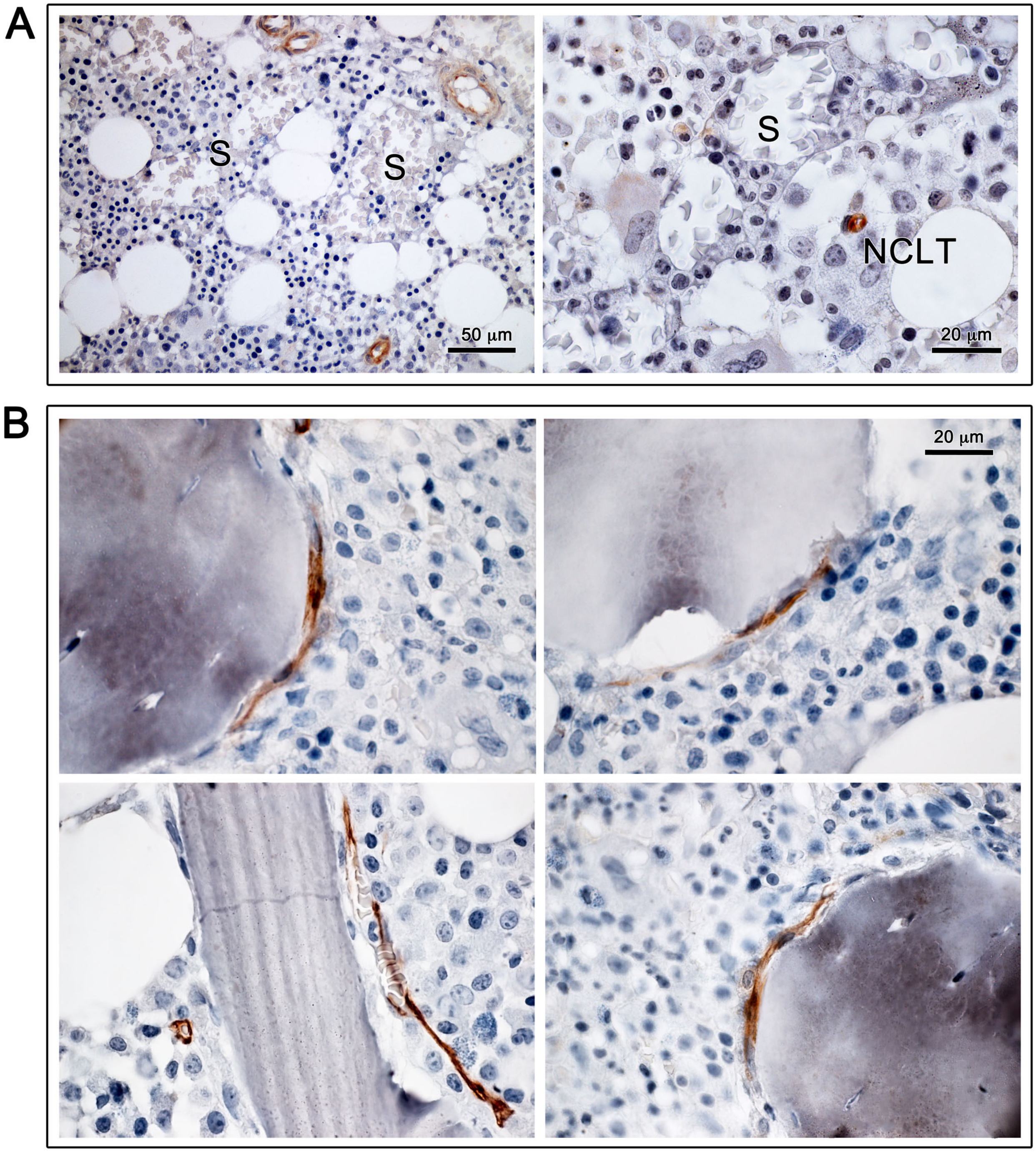

Supplemental Figure S3. (A) Convolute vessels with a large lumen area, characterized by a thin layer of endothelial cells and identified as sinusoids (S) resulted negative for NESTIN expression. (B) NESTIN^{+} capillary-like tubular structures (NCLTs) were frequently detected in direct contact with the bone trabeculae.

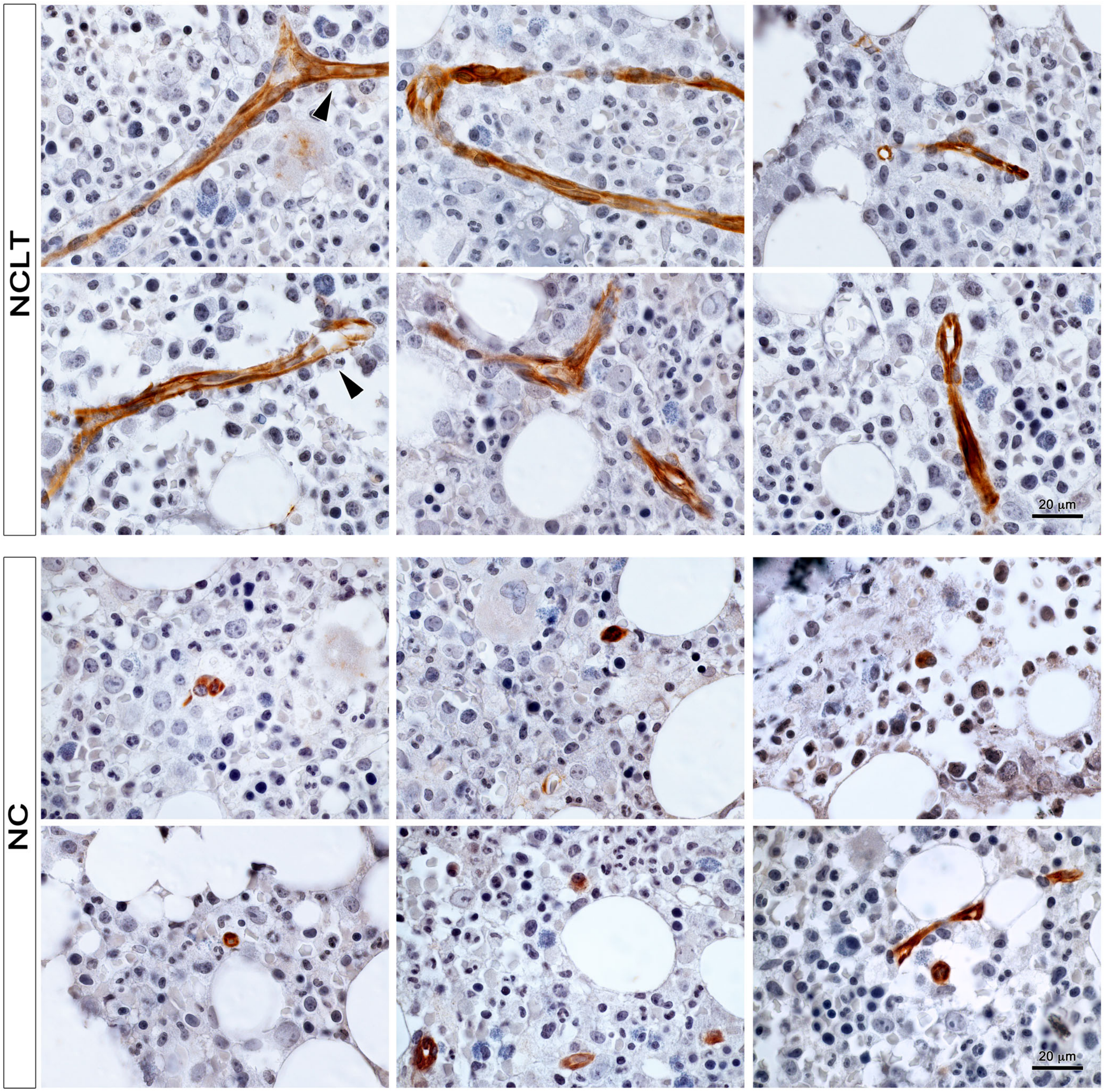

Supplemental Figure S4. NESTIN^{+} micro-vessels with imperceptible lumen described as NESTIN^{+} capillary-like tubes (NCLTs) are shown (upper panels). The capillary nature of NCLTs was suggested by frequent detection of compressed erythrocytes within the vessel walls (black arrow-head). Single or small clusters of NESTIN^{+} cells (NCs) were also detected (lower panels).

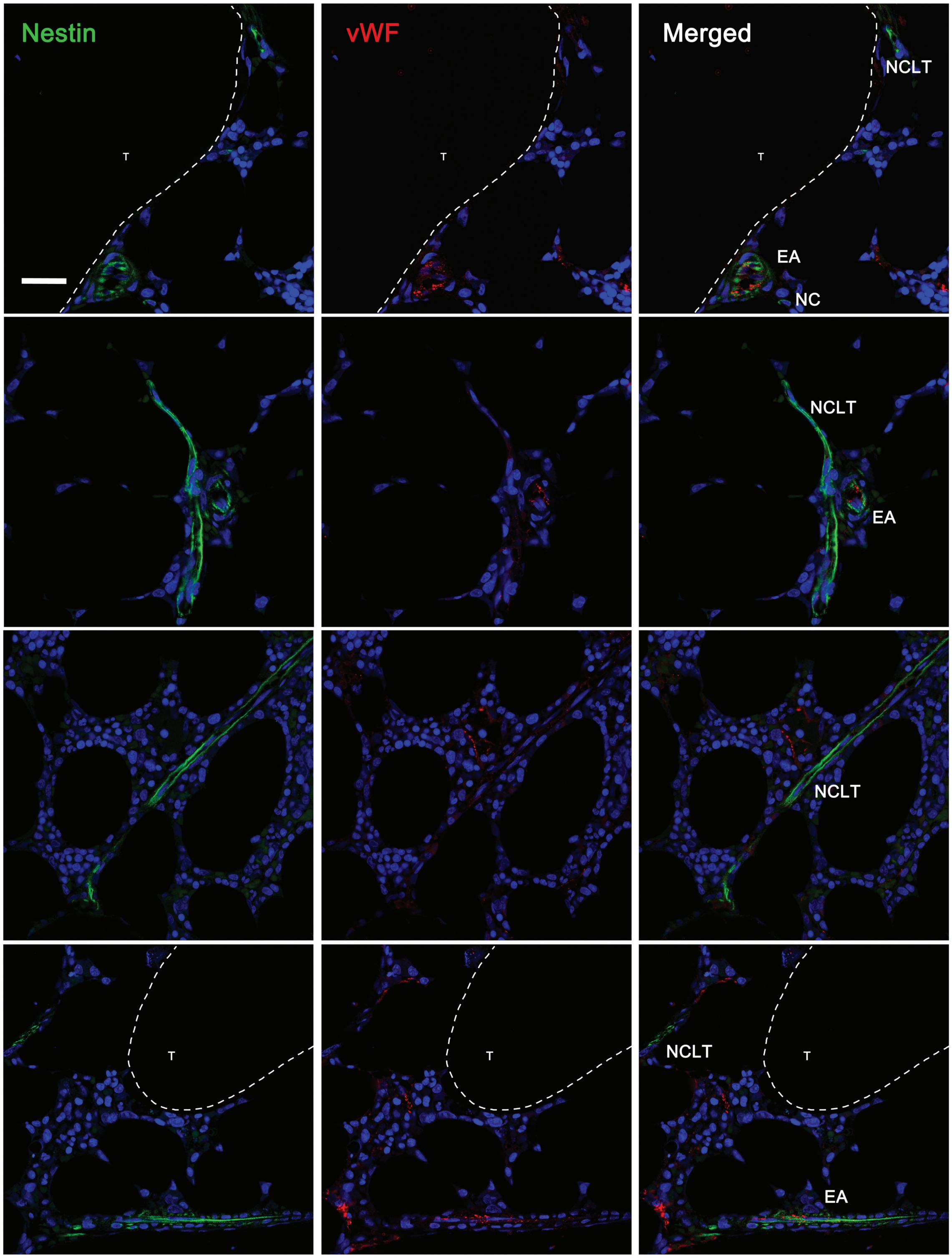

Supplemental Figure S5. *Three-colour immunofluorescence for Nestin and αSMA detection*. NESTIN$^+$ (green) arteries (*A*) and endosteal arterioles (EA) were encircled by a complex layer of perivascular cells or single pericytes as evidenced by αSMA positive stain (red), with a pattern similar to CD146. Lack of αSMA positivity around NCTLs and NCs confirmed the absence of associated perivascular cells. Nuclei were stained with DAPI (blue; scale bar: 20 μm).

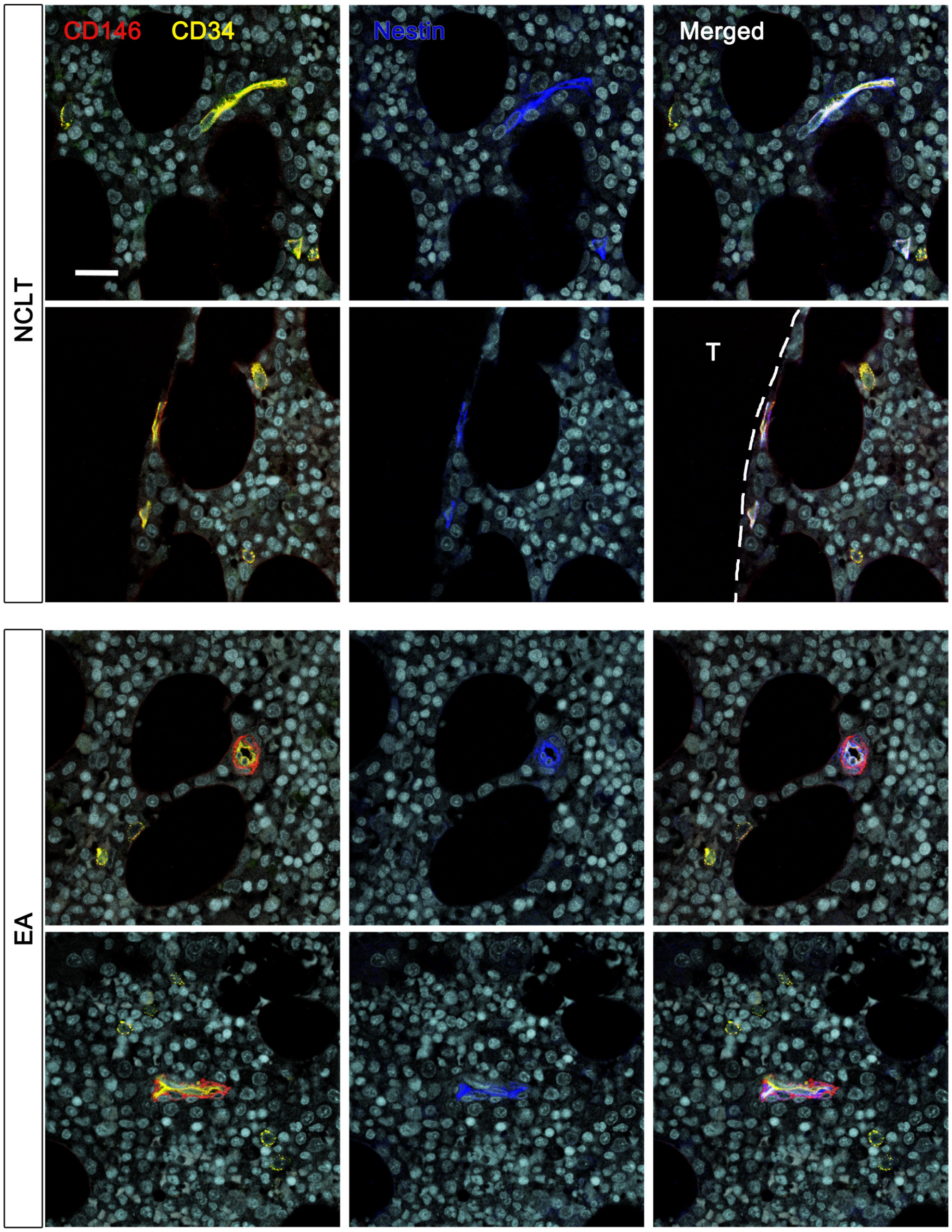

Supplemental Figure S6. <u>*Four-color immunofluorescence.*</u> The lack of high quality monoclonal antibodies produced in three different animal species to be applied for detection of three different markers has been partially overcame by "forced co-localization" staining method. This allowed simultaneous detection of CD146 (red), CD34 (yellow, resulting from the forced co-localization of green and red signals) and NESTIN (blue). This particular staining method and pseudo-color palette, showed the $CD34^{+}NESTIN^{bright}$ endothelial wall in brilliant white as consequence of markers overlap. The dotted pattern yellow fluorescence easily allowed to discriminate imHCs in proximity of NESTIN-positive micro-vessels that can be discriminate in NCLT or EA depending on the presence of perivascular cells marked in red. Nuclei stained by DAPI are in cyan pseudocolor. (T = bone trabecula, scale bar: 20 μm).

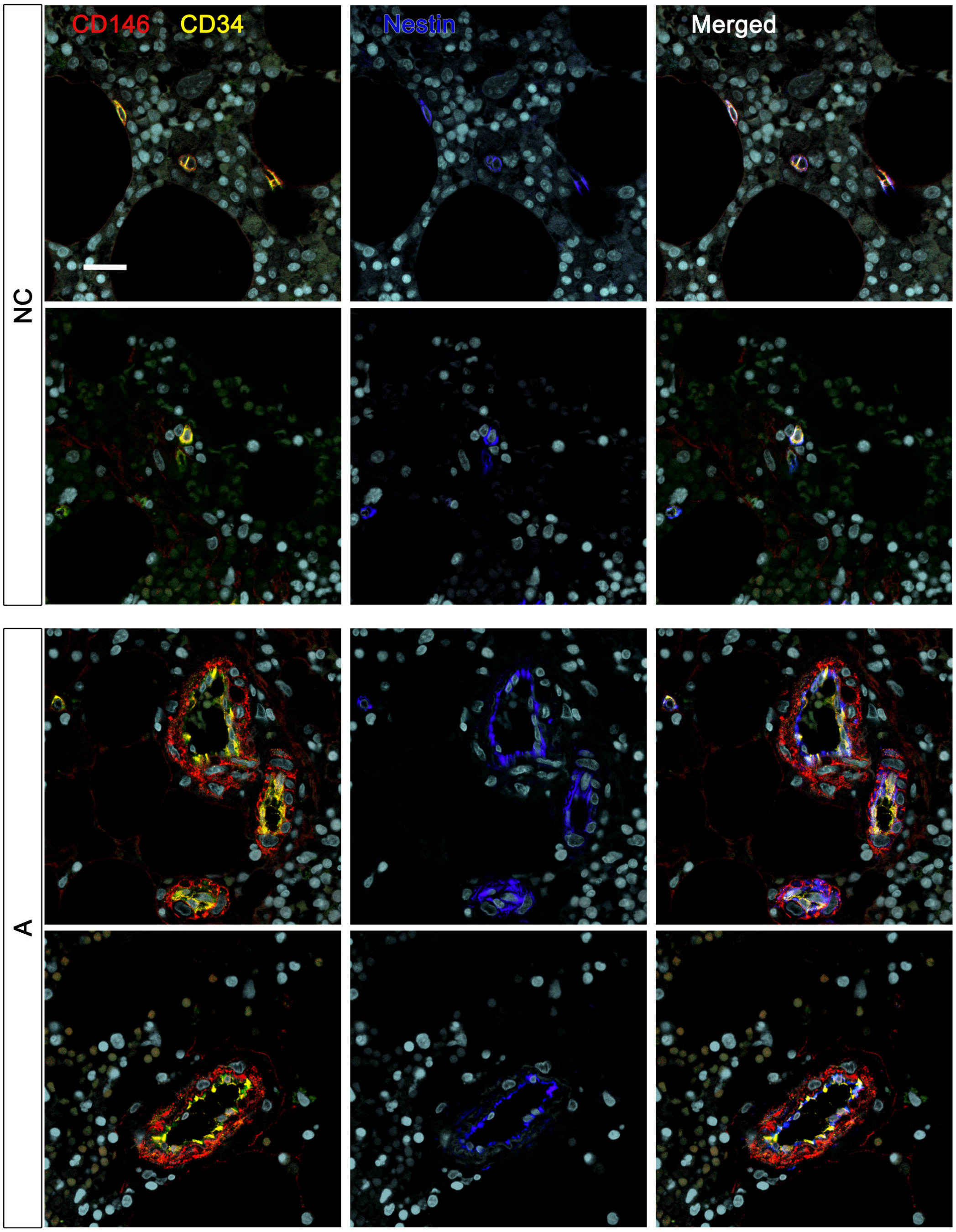

Supplemental Figure S7. *Four-colour immunofluorescence* for CD146 (red), CD34 (yellow), and NESTIN (blue) of NCs (upper panels) and arteries (A, lower panels). Nuclei were stained with DAPI (cyan; scale bar: 20 µm)

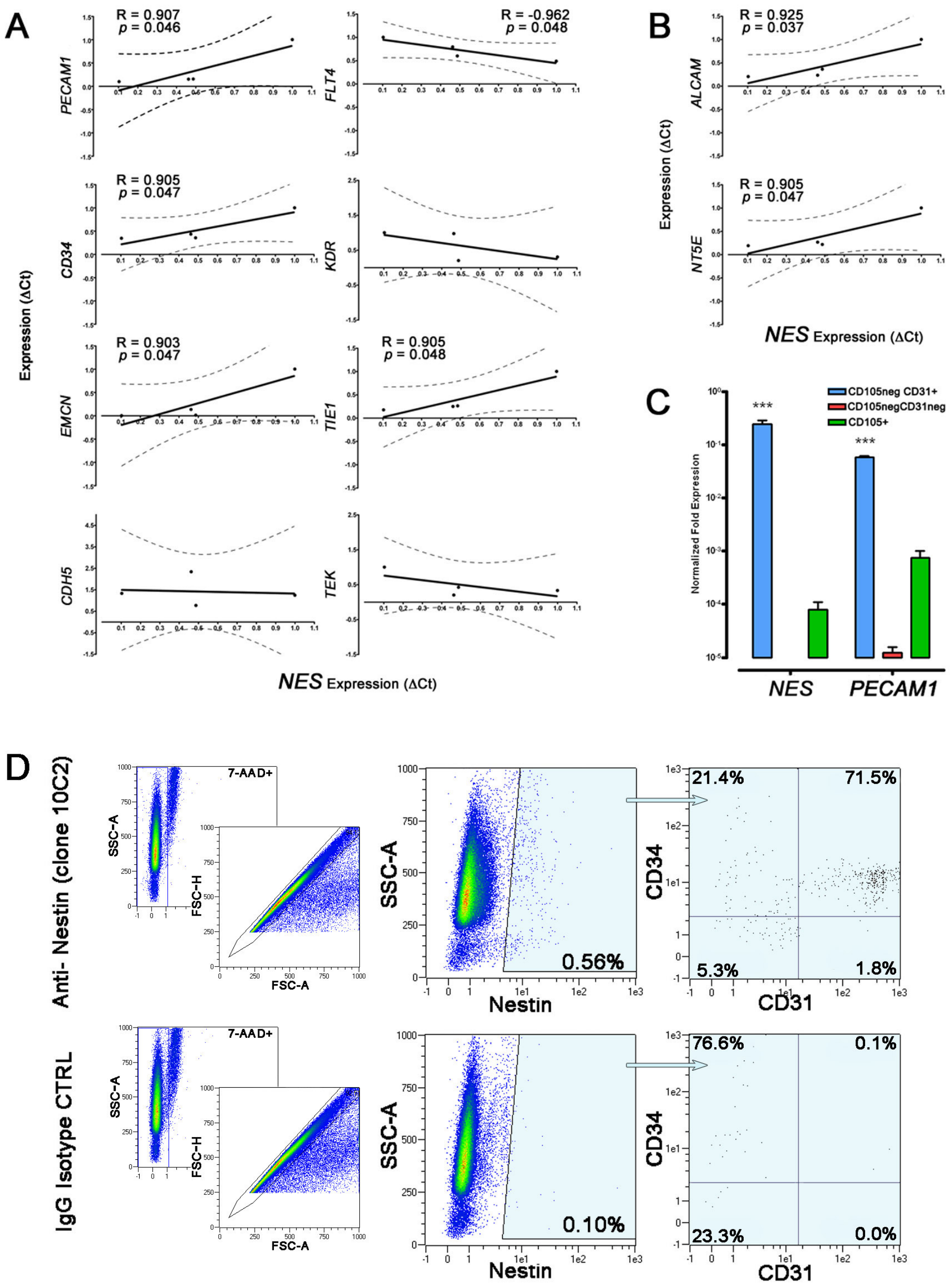

Supplemental Figure S8. (A) In BM-derived nucleated cells, *NES* expression positively correlated with *PECAM1* (CD31), *CD34*, *EMCN* (Endomucin) and *TIE1* (Angiopoietin-1 receptor) BMEC markers expression but negatively correlated with *FLT4* (VEGFR-3) specifically expressed by sinusoidal BMECs. (B) Positive correlation has been also detected among *NES* and *ALCAM* (CD166) and *NT5E* (CD73) MSC markers. (C) *NES* has been also detected in $CD105^{neg}CD31^{+}$ immune-magnetically sorted BM population (pale blue bars) and significantly at lower levels in $CD105^{+}$ fraction (green bars), no expression was reported for the double negative population (red bars, *** $p<0.001$). (D) Flow cytometry revealed that most of the rare NESTIN-positive cells express CD34 and CD31 surface markers. Doublets and 7-AAD-positive dead cells were excluded before gating for NESTIN-positive events.

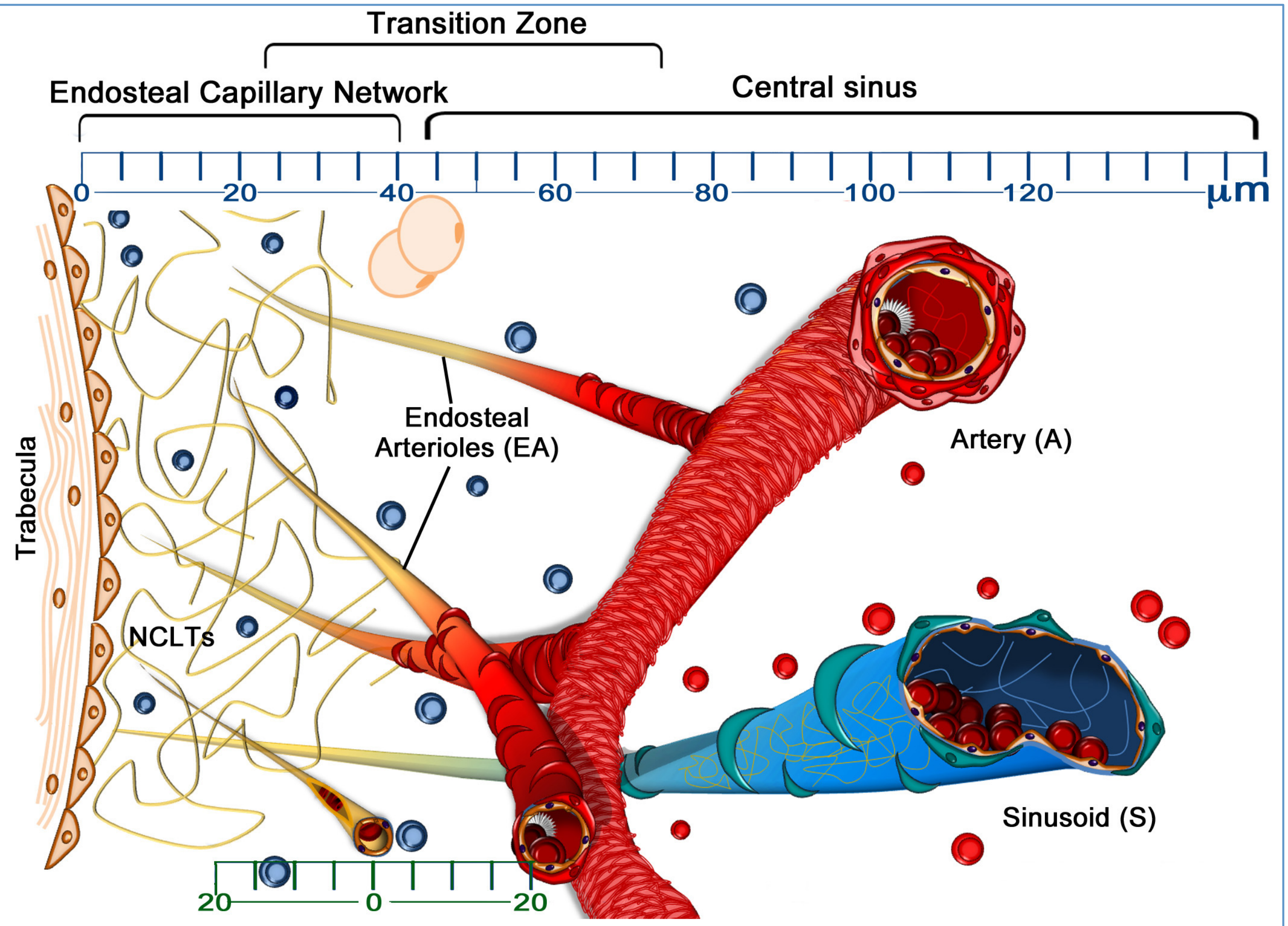

	NCLTs	EA	A	S
BMEC CD34	Bright	Bright	Bright	Dim
BMEC Nestin	Bright	Bright	Bright	-
BMEC Nuclear morphology	Elongated	Elongated	Elongated	Rounded
Inner calibre (µm)	≤ 4.5	4.6 - 7.5	> 7.6	> 20
2nd layer of perivascular cells	N.D.	Single layered $Nes^{dim}CD146^{+}\alpha SMA^{+}$	Complex multilayered $Nes^{dim}CD146^{+}\alpha SMA^{+}$	N.D
Distance to the endosteum	0 - 10 µm<br>11- 40 µm	0- 40 µm	Random	N.D

Supplemental Figure S9. Schematic representation of the "endosteal capillary niche" hypothesis. Oxigenated blood enters the BM vascular network by arteries (red vessels) and it is drained out into the central sinus through sinusoids (blue vessels). Differences in their morphology, complexity and wall permeability led to the hypothesis of different roles in HSPC regulation in the perivascular niche. Distinctive expression of CD34 and NESTIN in arterial *vs* sinusoidal BMECs has been indeed reported. Nearing the endosteum, arteries branch into smaller endosteal arterioles that reconnect to the downstream sinusoids by a complex capillary network in the so called "transition zone". The existence of a relationship between HSPCs (blue HSPCs) and NCLTs could be hypothesized because of $CD34^{+}$ cell proximity (<20 µm) to osteoblasts and bone-lining cells. (BMEC: bone marrow endothelial cells, N.D.: not detected)

Supplemental Video 1. 3D reconstruction of a *NCLT* from confocal laser scanning image stacks reveals the micro-tubular structure and an empty lumen. Nestin expression in green, DAPI nuclear stain in blue.

Supplemental Video 2. 3D reconstruction of a *NC* from confocal laser scanning image stacks. Even if slightly elongated, the NESTIN-expression (green) encircles a DAPI+ nucleus (blue) without break of continuity.
